# Metformin transiently inhibits colorectal cancer cell proliferation as a result of either AMPK activation or increased ROS production

**DOI:** 10.1038/s41598-017-16149-z

**Published:** 2017-11-22

**Authors:** Angela Mogavero, Maria Valeria Maiorana, Susanna Zanutto, Luca Varinelli, Fabio Bozzi, Antonino Belfiore, Chiara C. Volpi, Annunziata Gloghini, Marco A. Pierotti, Manuela Gariboldi

**Affiliations:** 1 Department of Experimental Oncology and Molecular Medicine, Fondazione IRCCS Istituto Nazionale dei Tumori, via G. Amadeo 42, 20133 Milan, Italy; 2 Molecular Genetics of Cancer, Fondazione Istituto FIRC di Oncologia Molecolare, via Adamello 16, 20139 Milan, Italy; 3 Department of Diagnostic Pathology and Laboratory Medicine, Fondazione IRCCS Istituto Nazionale dei Tumori, via G. Venezian 1, 20133 Milan, Italy

## Abstract

Metformin is a widely used and well-tolerated anti-diabetic drug that can reduce cancer risk and improve the prognosis of certain malignancies. However, the mechanism underlying its anti-cancer effect is still unclear. We studied the anti-cancer activity of metformin on colorectal cancer (CRC) by using the drug to treat HT29, HCT116 and HCT116 p53−/− CRC cells. Metformin reduced cell proliferation and migration by inducing cell cycle arrest in the G0/G1 phase. This was accompanied by a sharp decrease in the expression of c-Myc and down-regulation of IGF1R. The anti-proliferative action of metformin was mediated by two different mechanisms: AMPK activation and increase in the production of reactive oxygen species, which suppressed the mTOR pathway and its downstream targets S6 and 4EBP1. A reduction in CD44 and LGR5 expression suggested that the drug had an effect on tumour cells with stem characteristics. However, a colony formation assay showed that metformin slowed the cells’ ability to form colonies without arresting cell growth, as confirmed by absence of apoptosis, autophagy or senescence. Our finding that metformin only transiently arrests CRC cell growth suggests that efforts should be made to identify compounds that combined with the biguanide can act synergistically to induce cell death.

## Introduction

The methods used for the early diagnosis of colorectal cancer (CRC) are insufficiently sensitive and specific and, despite major advances in surgical techniques and adjuvant treatment, there is still no effective therapy for advanced disease. About 50% of patients respond to the currently available systemic treatments, but almost all develop drug resistance; furthermore, targeted treatments are only effective in patients with a specific molecular profile, and these are still at very high risk of developing resistant mutations. There is therefore growing interest in finding alternative treatments.

Metformin (1,1-dimethylbiguanide hydrochloride) is frequently prescribed to reduce hepatic gluconeogenesis and increase skeletal muscle glucose uptake in patients with type 2 diabetes. It also directly inhibits the growth of various tumour types *in vitro* and *in vivo* ^1^, and seems to reduce the growth of tumour initiating cells or cancer stem cells (CSCs)^2^. The inhibitory effect of metformin may be mediated by the inactivation of the mammalian target of rapamycin (mTOR), mainly as a result of the activation of adenosine monophosphate-activated protein kinase (AMPK), changes in intracellular reactive oxygen species (ROS) levels^3^, the inhibition of mitochondrial functions^4^, or by various other mechanisms that have been associated with the drug^1^.

Earlier *in vitro* studies demonstrated that metformin can inhibit the proliferation of CRC cells^5^, and *in vivo* studies have shown that metformin delays tumour onset in a mouse model of *TP53* mutant CRC^6^ and inhibits the growth of colon carcinomas stimulated by a high-energy diet^7^. Consequently, a number of clinical trials are investigating the effect of metformin on CRC in humans. The results of some of these suggest that it has anti-tumour activity and improves overall survival^8–10^, but others have come to opposite conclusions. Tsilidis *et al*.^11^ compared metformin with sulfonylurea, and did not find that the biguanide was associated with a reduced cancer risk in a large cohort of patients with newly diagnosed diabetes, and a three-year study of patients with stage II CRC found no significant relationship between metformin treatment and the risk of cancer recurrence or death^12^.

We analysed the activity of metformin in three CRC cell lines, one with BRAF as driver mutation, the others with KRAS mutation, one of which is an isogenic p53 null form and found for the first time that the drug transiently inhibited their growth and mTOR pathway activation by means of AMPK-dependent and -independent mechanisms.

## Results

### Metformin reduces cell proliferation and motility

**Figure 1:**
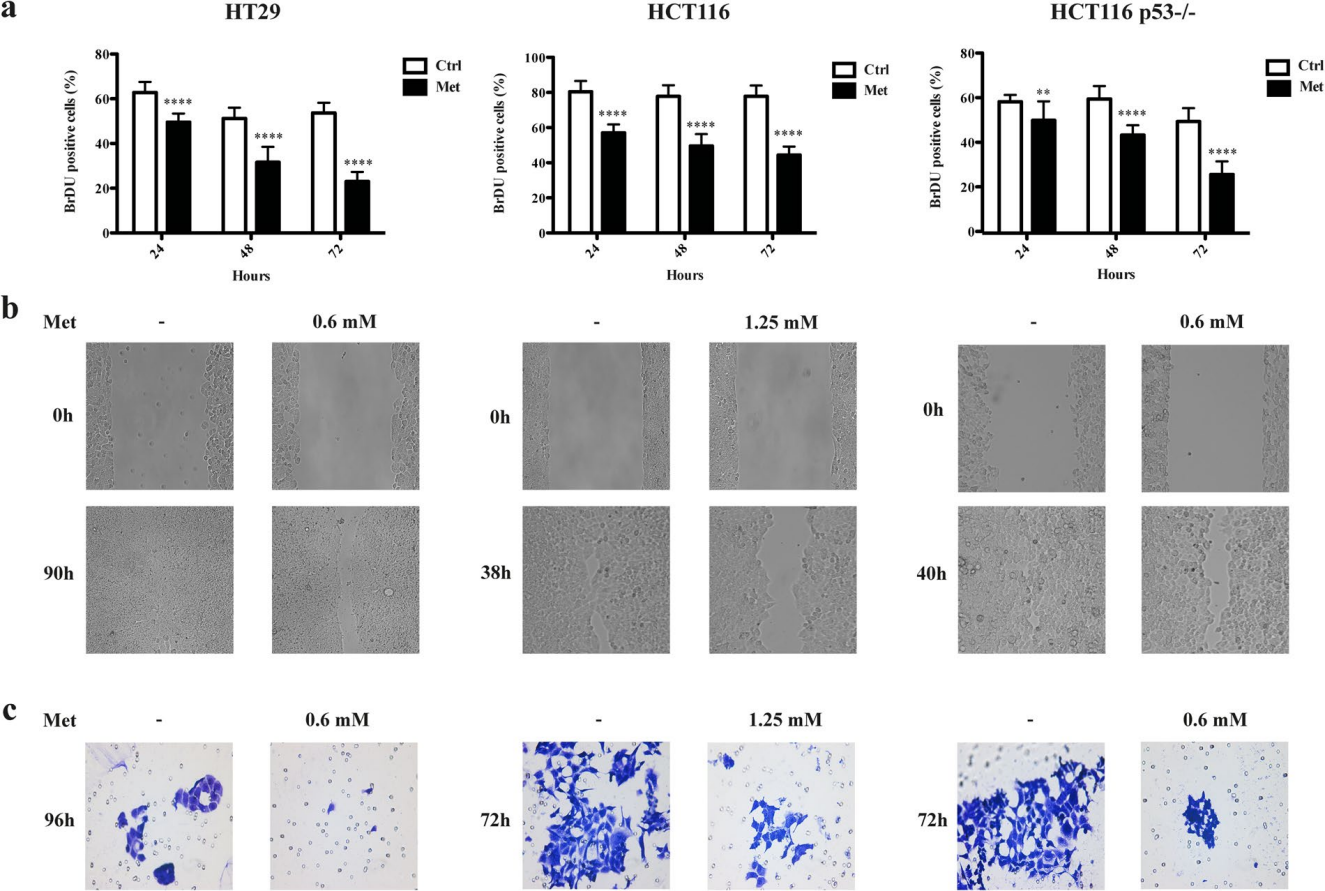
Metformin (Met) decreased the proliferation, migration, and invasion of HT29, HCT116 and HCT116 p53−/− cells. (**a**) Cell proliferation was evaluated *in vitro* by means of BrdU incorporation in the absence (Ctrl) or presence of 5 mM Met after 24, 48 and 72 hours’ treatment. The results are shown as mean values ± SD compared with the control group (**P < 0.01, ****P < 0.0001). (**b**) The wound healing assay was conducted after Met treatment (0.6 mM for HT29 and HCT116 p53−/−; 1.25 mM for HCT116) for 90 hours (HT29), 38 hours (HCT116) or 40 hours (HCT116 p53−/−). (**c**) The chamber invasion assay was performed after treatment with 0.6 mM or 1.25 mM Met for 96 hours (HT29) or 72 hours (HCT116 and HCT116 p53−/−).

In order to investigate the effects of metformin on CRC, the HT29, HCT116 and HCT116 p53−/− cell lines were treated with 5 mM metformin as reported by Zakikhani *et al*.^5^. As shown in Fig. 1a and in Supplementary Fig. S1, MTT (3-(4,5-dimethylthiazol-2-yl)-2,5-diphenyltetrazolium bromide) and BrdU incorporation assays confirmed that this drug concentration inhibited cell growth. The decrease in proliferation (BrdU) after continuous exposure to metformin for 24, 48 and 72 hours was already detectable in all of the cell lines after 24 hours, and became more significant after 72 hours (from 54% to 23% in HT29, from 78% to 44% in HCT116, and from 50% to 26% in HCT116 p53−/− cells).

A modified wound scratch assay was used to assess the effects of metformin on the ability of CRC cells to migrate. Metformin was added at scalar concentrations ranging from 5 to 0.3 mM, derived from the MTT assays and including non-cytostatic doses of the drug (Supplementary Fig. S2). In untreated HT29 cells, wound closure was complete within 90 hours (Fig. 1b); in the presence of 0.6 mM metformin, migration was less and wound closure occurred more than 96 hours after treatment. Untreated HCT116 and HCT116 p53−/− cells migrated more quickly, and the wound was closed in respectively 38 and 40 hours (Fig. 1b and Supplementary Fig. S2); in the presence of 1.25 mM (HCT116 cells) and of 0.6 mM (HCT116 p53−/−) metformin, it took respectively 43 and 45 hours.

Finally, a matrigel chamber invasion assay showed that metformin inhibited tumour invasion in the three cell lines at all the concentrations tested, but it was slower in the HT29 cells (Supplementary Fig. S3). Figure 1c displays results obtained at the same drug concentrations where a delay in migration was observed with the wound healing assay. This finding was supported by the reduction in matrix metalloproteinase 9 (MMP9) mRNA expression^13^ in the HCT116 and HCT116 p53−/− cells (Supplementary Fig. S1), while HT29 cells do not express MMP9^14^.

### Metformin increases the percentage of cells in the G0/G1 phase, reduces the expression of cyclin D1 and c-Myc and the phosphorylation of Rb

**Figure 2:**
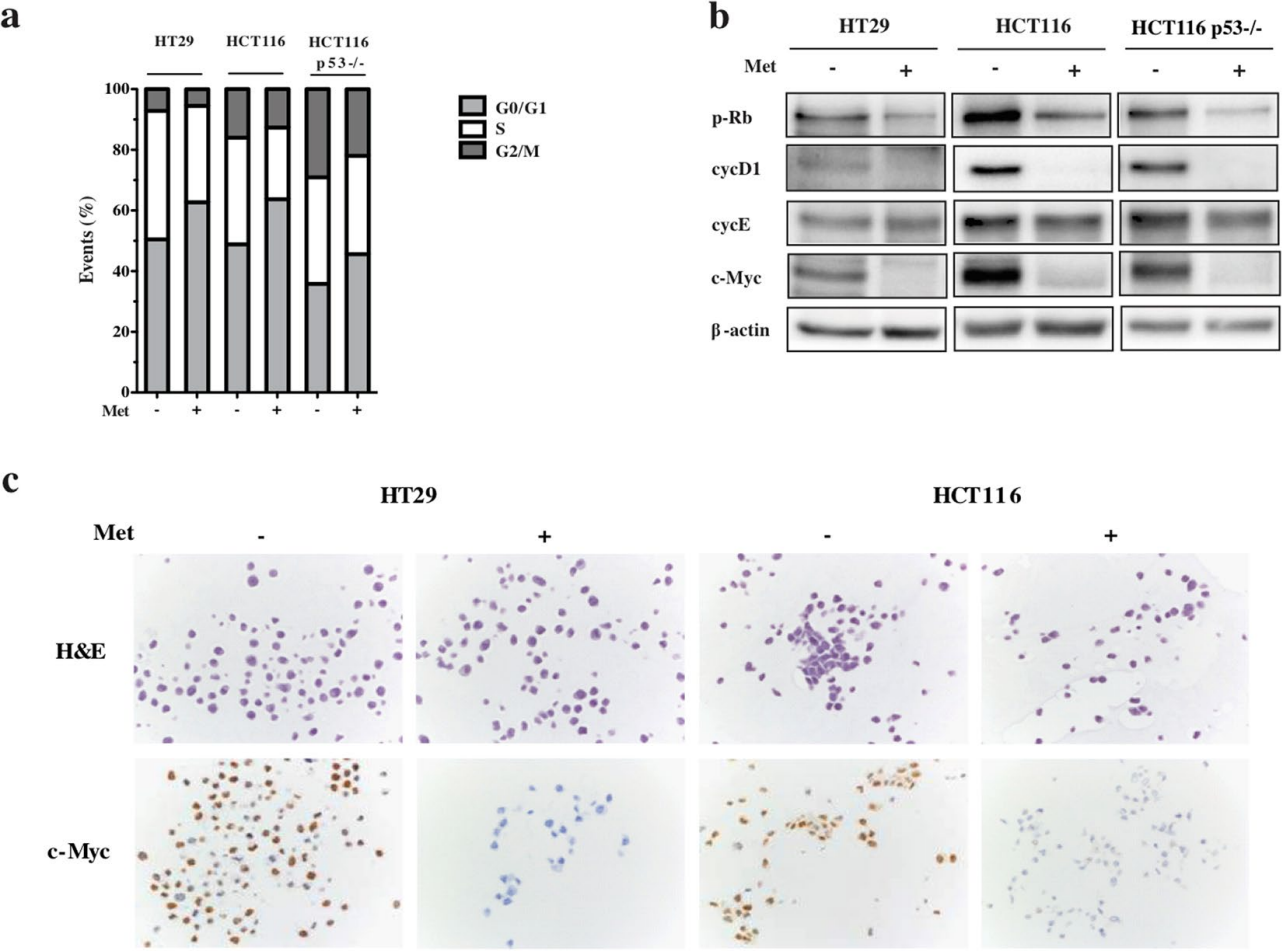
Metformin (Met) increases the percentage of cells in the G0/G1 phase, and affects the expression of various cell cycle regulatory proteins in HT29, HCT116 and HCT116 p53−/− cells. (**a**) Flow cytometric analysis of proliferating cells 72 hours after the addition of 5 mM Met. The results are representative of three independent experiments. (**b**) Immunoblots of p-Rb, cycD1, cycE and c-Myc in cells treated for 72 hours with 5 mM Met or left untreated. β-actin was used as the loading control. Full-size blots are shown in Supplementary Fig. S10. (**c**) Immunohistochemistry of c-Myc.

In order to investigate the cell mechanisms reducing proliferation, we cytometrically evaluated the changes in cell cycle progression induced by metformin. After 72 hours of treatment, there was a slight accumulation of cells in the G0/G1 phase (from 50% to 63% of HT29 cells, from 49% to 64% of HCT116 cells, and from 36% to 46% of HCT116 p53−/− cells), and a corresponding decrease in the percentage of cells in the G2 phase (from 7.17% to 5.52% of HT29 cells, from 16.02% to 12.69% of HCT116 cells, and from 29.11% to 21.99% of HCT116 p53−/− cells) in comparison with the untreated cells (Fig. 2a).

Immunoblotting analysis of cyclins D1 and E (which are involved in the G1-S transition) confirmed the increase in the percentage of cells in the G0/G1 phase. Cyclin D1 (cycD1) was significantly down-regulated in all three cell lines, and there was no change in cyclin E (cycE). In line with the inactivation of cycD1, metformin decreased retinoblastoma protein (pRb) phosphorylation (a cyclin D1 downstream target) and c-Myc expression (Figs. 2b and c). Finally, IHC revealed a considerable decrease in histone H3 phosphorylation (P-HH3) staining (Supplementary Fig. S1).

### Metformin does not induce apoptosis, autophagy or senescence

In order to investigate the biological mechanisms underlying the metformin-induced inhibition of cell proliferation, we evaluated the drug’s ability to induce CRC cell death by means of apoptosis, autophagy or senescence.

**Figure 3:**
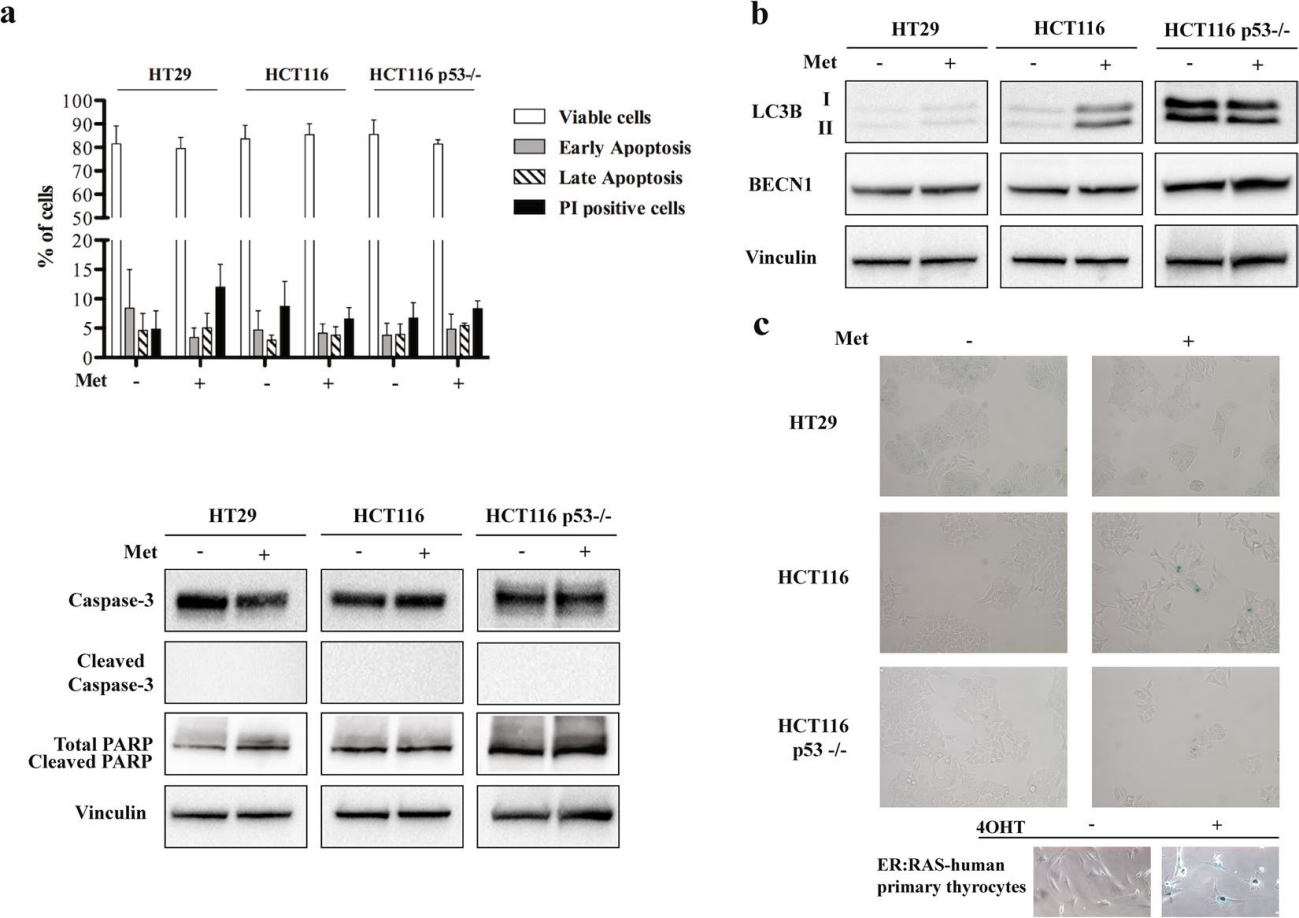
Metformin (Met) does not induce apoptosis, autophagy and senescence in HT29, HCT116 and HCT116 p53−/− cells. (**a**) Graphic representation of the results obtained from Annexin V assay (upper panel) and western blot analysis of Caspase-3 and PARP activation after treatment with 5 mM Met for 72 h (lower panel). Vinculin was used as the loading control. (**b**) Immunoblotting determination of LC3B and BECN1 protein after treatment with 5 mM Met for 72 hours; Vinculin was used as the loading control. Full-size blots are shown in Supplementary Fig. S11. (**c**) β-galactosidase staining of untreated cells (−) and cells treated with Met for 72 hours (+). The images were acquired at 20x magnification. At the bottom, human primary thyrocytes carrying the ER:RAS vector were left untreated or treated with 200 nM 4-hydroxytamoxifen (4OHT) for seven days, and used as positive controls (magnification 40x). The data are representative of at least three independent experiments.

Annexin V assay showed no induction of apoptosis after 72 hours of treatment (Fig. 3a), and this was confirmed by the absence of poly (ADP-ribose) polymerase protein (PARP) and of Caspase-3 cleavage (Fig. 3a) as well as from the Caspase-3 FACS analyses (Supplementary Fig. S4).

Immunoblotting analysis of the light chain 3 isoforms I and II (LC3BI and LC3BII) showed that treatment with metformin did not induce a conversion from the LC3-I to the LC3-II form (Fig. 3b), typical of autophagy. Immunofluorescence for LC3B (Supplementary Fig. S5) and also BECN1 expression did not vary after treatment with metformin in all the cell lines analysed (Fig. 3b), suggesting the absence of autophagy. However, the finding that metformin increased both LC3B isoforms in HCT116 cells might support an induction of autophagy in this cell line as described by Buzzai *et al*.^6^. Finally, the fact that β-galactosidase staining showed no differences between untreated and metformin-treated cells of all three cell lines excluded the activation of senescence (Fig. 3c).

### Metformin-induced inhibition of proliferation is reversible

**Figure 4:**
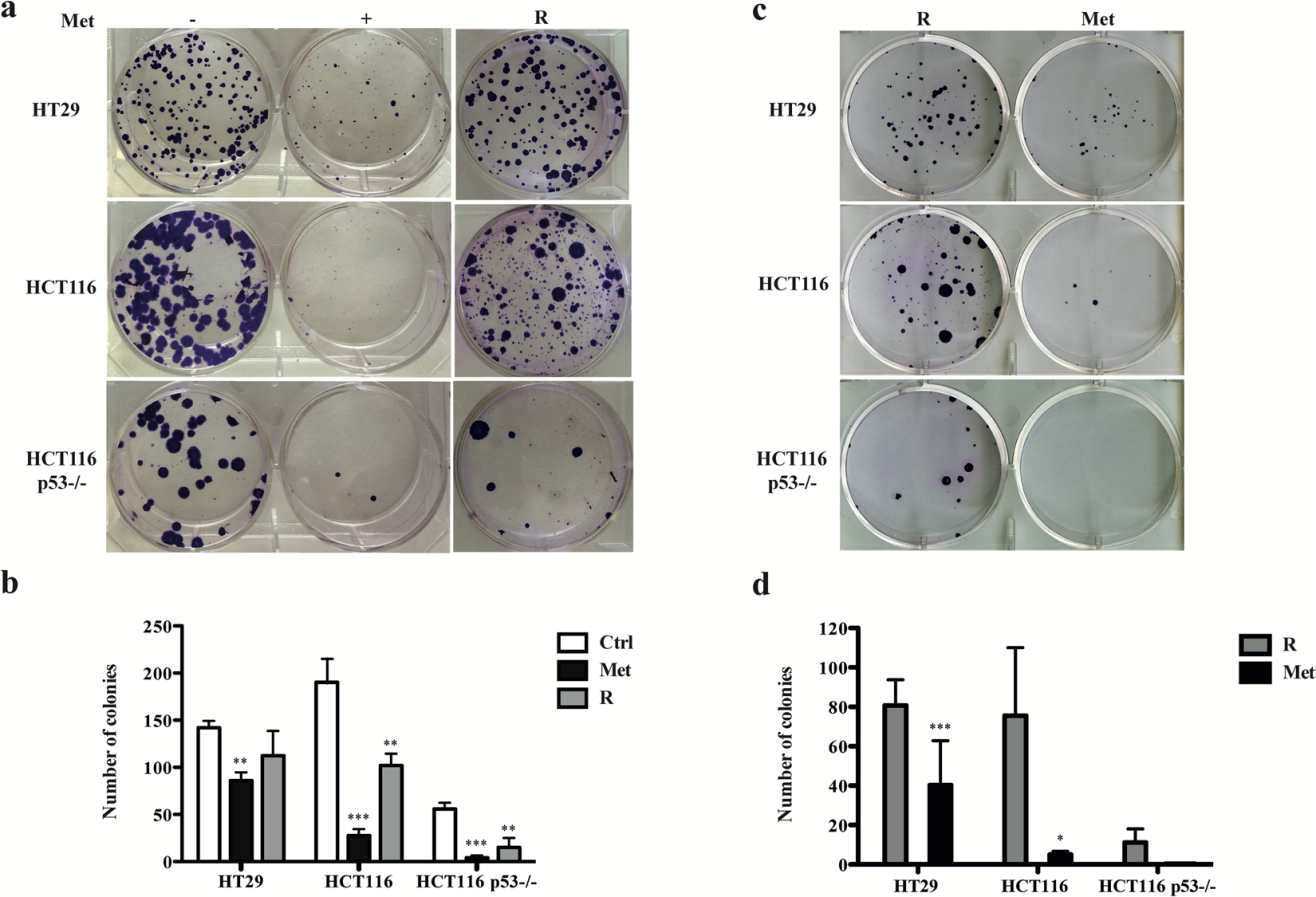
Metformin (Met) reversibly inhibits colony formation. (**a**) One hundred cells were grown for 12 days in 6-well plates with or without Met (5 mM). The “rescued” colonies (R) are cells treated with Met for 12 days and grown for a further 12 days in fresh complete medium without Met. (**b**) Graphic representation of the results. The bars indicate the mean value ± SD of three independent experiments (**P < 0.01, ***P < 0.001). (**c**) One hundred “rescued” cells (R) were grown for 12 days in 6-well plates in presence or absence of Met. (**d**) Graphic representation of the results. The bars indicate the mean value ± SD of three independent experiments (*P < 0.05, ***P < 0.001).

To further investigate the absence of induced cell death after metformin treatment, we examined the clonogenic capacity of the three cell lines. Six days’ treatment with metformin significantly reduced the number and size of colonies in comparison with the untreated controls (Supplementary Fig. S6). This was confirmed after 12 days, and was maintained for up to 18 days of treatment (Fig. 4 and Supplementary Fig. S6). The ability to form colonies was least in the two HCT116 cell lines. After drug removal and its replacement with complete medium, the “rescued cells” resumed proliferating at all time points (Fig. 4 and Supplementary Fig. S6). Although this recovery was less marked in the HCT116 p53−/− cell line, this effect does not seem to depend on *TP53* status, which is mutated also in HT29 cells. Treatment of “rescued cells” with metformin for 12 days reduced the number and size of the colonies, in comparison with the untreated control and, again, the ability to form colonies was least in the two HCT116 cell lines (Fig. 4). These results are also supported by the finding that the viability and the proliferation of metformin treated cells are comparable to those of rescued cells cultured for 6 days in fresh medium and then treated with metformin for 3, 6 and 12 days (see Supplementary Figs. S7 and S8). Finally, the expression patterns of p-Rb, cycD1, cycE and c-Myc and of untreated and “rescued cells” are similar (Supplementary Fig. S9).

These findings are in line with the absence of cell death, and suggest that metformin has an inhibitory effect on CRC cell proliferation that can be overcome after drug removal.

### Metformin inhibits the mTOR pathway as a result of different mechanisms

**Figure 5:**
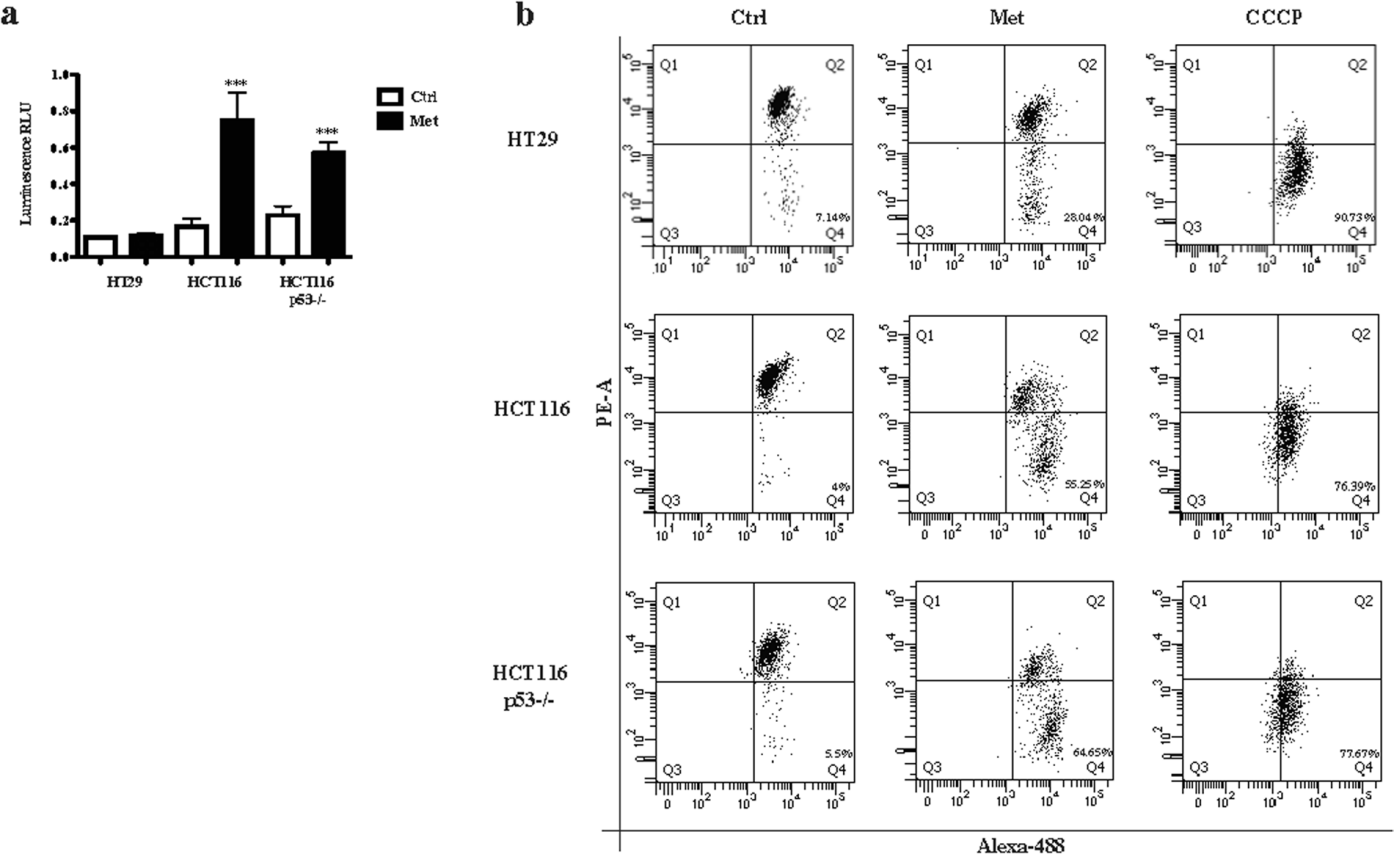
Metformin (Met) causes oxidative stress and induces mitochondrial depolarization. The cells were treated with 5 mM Met for 72 hours and a significant increase in ROS production was measured in HCT116 and HCT116 p53−/− cell lines. The bars represent the mean value ± SD of three independent experiments (***P < 0.001) (**a**). In line with ROS increase, HCT116 and HCT116 p53−/− also showed a stronger mitochondrial depolarisation respect to HT29 (**b**). Mitochondrial depolarization was revealed by decrease of JC-1 PE and increase of JC-1 Alexa-488 signal intensities measured in the related cells. Before harvesting the cells, 1 μl of carbonyl cyanide 3-chlorophenylhydrazone (CCCP) was added as a positive control.

**Figure 6:**
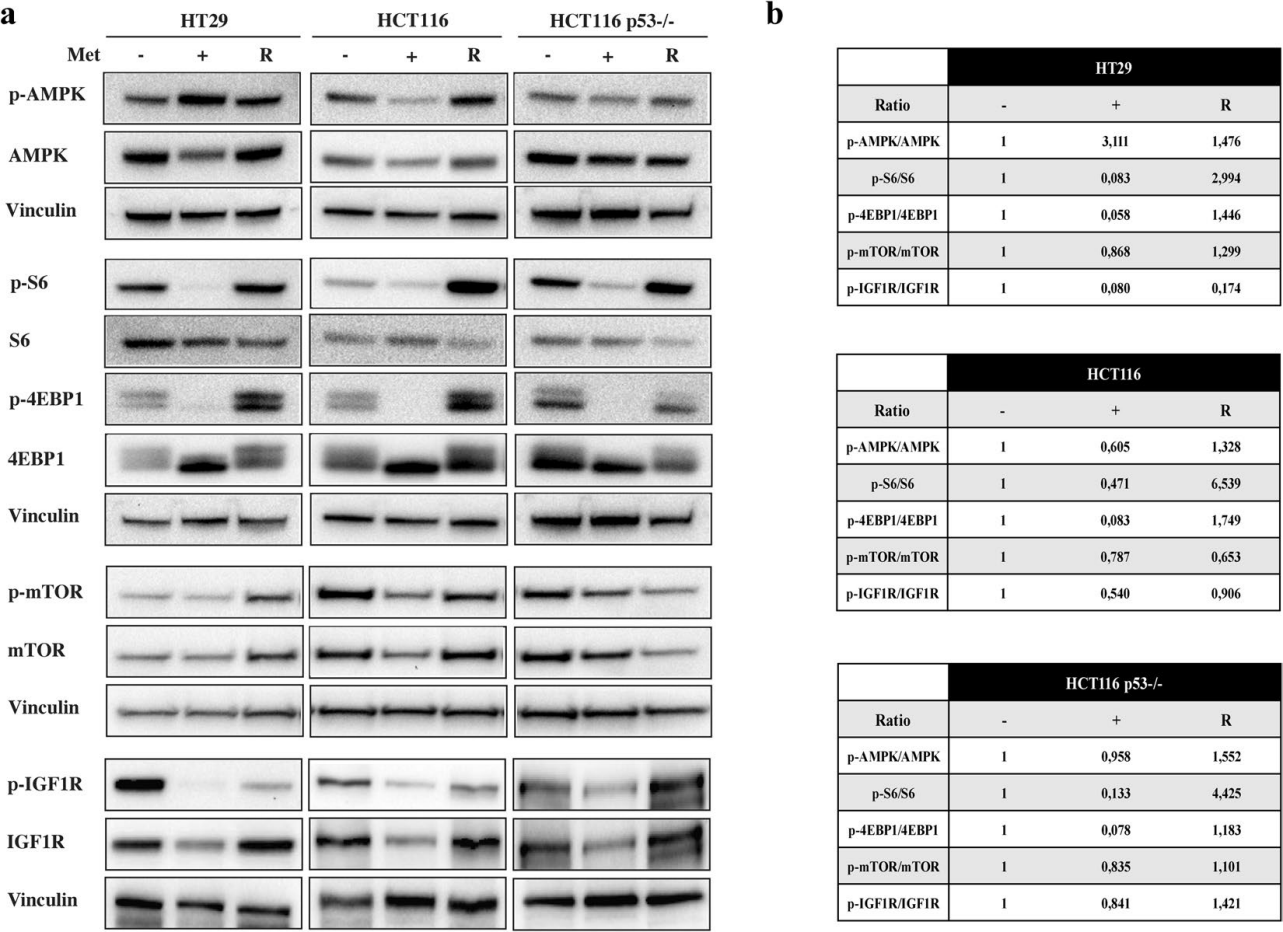
Metformin (Met) reversibly inhibits the mTOR pathway and IGF1R protein. (**a**) Immunoblots of cells treated with 5 mM Met for 72 hours, or left untreated. The “rescued” cells (R) were grown for 72 hours with Met and for a further 72 hours in fresh complete medium without Met. Vinculin was used as the loading control (in HCT116 and HCT116 p53−/− cells vinculin is the same for AMPK, S6 and 4EBP1 since they were analysed on the same blots). (**b**) Tables showing densitometric quantification of the western blot bands normalized to vinculin. The ratio of each phosphorylated protein to the corresponding total protein band was calculated and quantified with respect to the untreated sample (−) set to 1.0. The results are representative of three independent experiments. Full-size blots are shown in Supplementary Figs S12–S14.

At cellular level, metformin can inhibit mitochondrial respiratory complex I (CI)^15^, which leads to membrane depolarisation^16^, the release of ROS, and a decrease in the ATP/ADP ratio. The decrease in energy, resulting in AMPK activation, shuts down the mTOR/S6/4EBP1 axis and blocks protein synthesis^17^. After metformin treatment we observed respectively 3- and 2.5-fold increases in ROS production in the HCT116 and HCT116 p53−/− cells, but none in the HT29 cells (Fig. 5a). FACS analysis and JC-1 staining were used to investigate changes in mitochondria membrane potential. After 72 hours of metformin treatment, there was a significant shift in fluorescence, with a decrease in PE and an increase in Alexa-488 emission, indicating mitochondria depolarisation in the HCT116 (55%) and HCT116 p53−/− cells (65%); in line with the absence of ROS production, this effect was weaker in HT29 cells (28.04%) (Fig. 5b). Metformin activated AMPK by phosphorylation of Thr172 only in the HT29 cells (in which the mitochondria were only marginally impaired by the drug). Despite these differences, metformin inhibited mTOR (Ser2448), ribosomal protein S6 kinase (RPS6K, Thr389) and 4E-binding protein 1 (4EBP1, Thr37/46) in all of the cell lines (Fig. 6).

Taken together, these findings indicate that inhibition of the mTOR/S6/4EBP1 pathway in CRC cell lines is only partially mediated by mitochondrial depolarisation and AMPK activation, and suggest that the cellular effects induced by metformin could be at least partially due to the mTOR-mediated reduction in protein synthesis. This view is supported by the recently published finding that inhibition of the mTOR/S6 branch significantly reduces the translation elongation step of protein synthesis in CRC mouse models^18^.

Interestingly, as previously found in other tumour histotypes^19,20^, metformin reduced the expression/phosphorylation (Tyr1135/1136) of IGF1R (Fig. 6), the activation of which induces the proliferation, differentiation and transformation of cancer cells^21^.

It is particularly interesting to note that drug removal reduced the phosphorylation of AMPK in HT29 cells and increased mTOR/S6/4EBP1/IGF1R phosphorylation profile in all “rescued” samples, except for mTOR in HCT116 cells (Fig. 6), thus confirming that the metformin-induced blockade of cell proliferation is transient.

### Metformin reduces the expression of CRC stem cell markers

**Figure 7:**
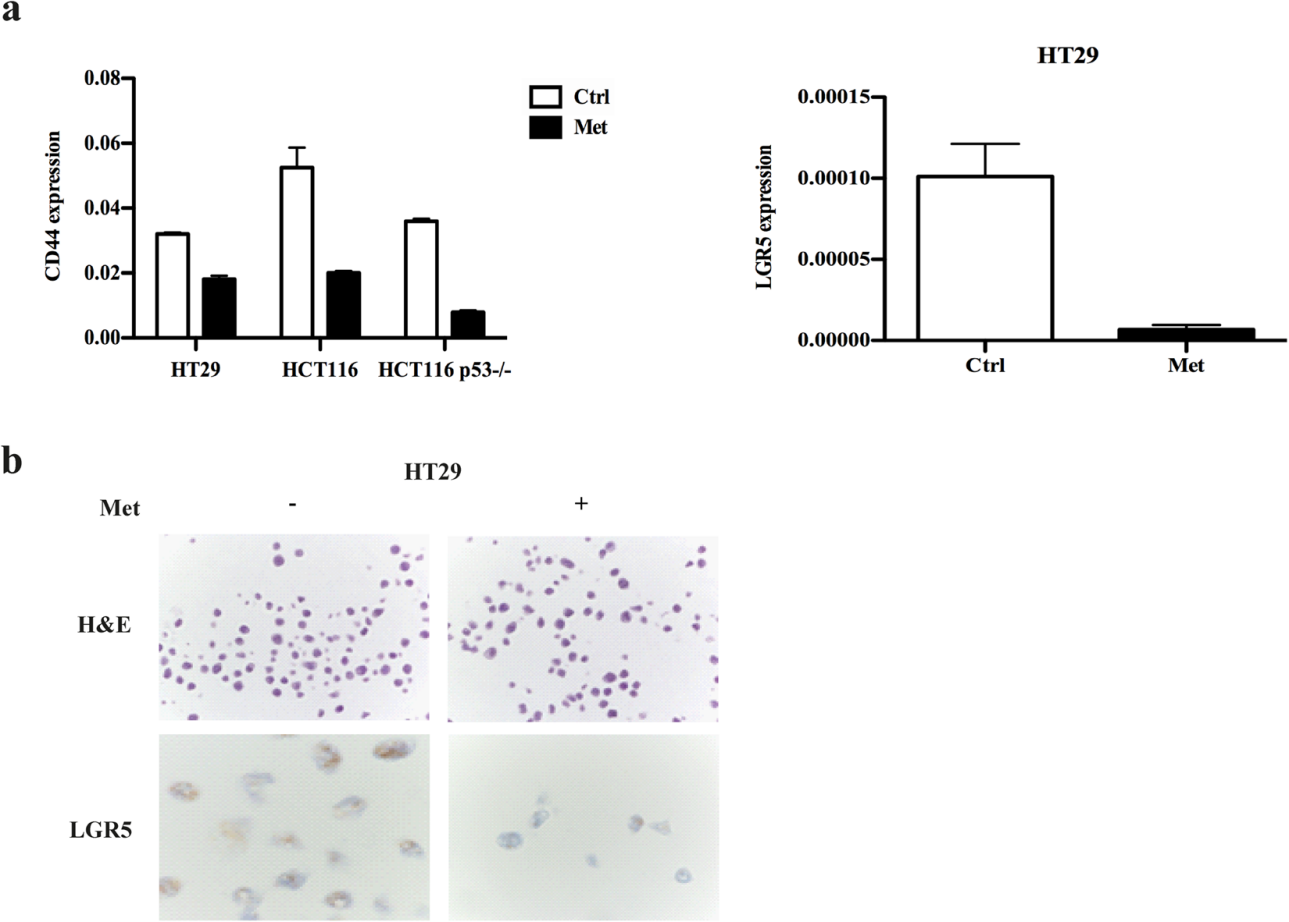
Metformin (Met) affects the expression of the CRC stemness markers LGR5 and CD44. (**a**) qRT-PCR of CD44 and LGR5 in HT29, HCT116 and HCT116 p53−/− cells. (**b**) *In situ* hybridisation of LGR5 in HT29 cells. Hematoxylin and eosin (H&E) staining is the same as in Fig. 2.

In addition to promoting cell proliferation, c-Myc is highly expressed in cancer cells with stem cell properties^22^, and the sharp reduction in c-Myc expression after treatment with metformin suggests that the drug has an effect on the stem cell component of CRC. We investigated this possibility by evaluating the expression of the CRC stem cell markers CD44 and LGR5, and found that metformin treatment reduced CD44 mRNA levels in all the cell lines (Fig. 7a, left). The biguanide also reduced LGR5, which was expressed only in HT29 cells^23^, as observed both by qRT-PCR (Fig. 7a, right) and ISH (Fig. 7b).

## Discussion

We investigated the *in vitro* effects of metformin on CRC cells because, although previous studies have shown that metformin inhibits the proliferation of CRC cells^5^, the drug’s underlying mechanisms of action on CRC are still unclear. We found that metformin was cytostatic and reduced cell motility, but did not induce cell death; furthermore, its effects were reversed after drug removal. As has been observed in other types of cancer^24–29^, the cytostatic effect of metformin on CRC cells is due to a reduction in cell proliferation, an increased accumulation of cells in the G0/G1 phase, a decrease in the expression of proteins controlling cell cycle progression such as cycD1 and c-Myc, and the inhibited phosphorylation of the cyclin D1 target pRb. Metformin reduced cell motility by decreasing cell migration and invasion, thus confirming what has been found in other types of cancer^20,30^. We also observed a decrease in the mRNA expression of MMP9, a matrix metalloproteinase expressed in CRC^31^, and a reduction in the mRNA expression of the two established cancer stem cell (CSC) markers CD44 and LGR5^32,33^.

There are no published findings showing that metformin induces apoptosis in CRC, and the absence of cell mortality after treatment with the drug has previously been observed in prostate and breast cancer cells^24,34^. It has been suggested that metformin may only induce apoptosis when cells are treated almost at confluence^35^. We worked under low density conditions (5 × 10^4^ cells/cm^2^) because the drug induced a high degree of acidosis (a decrease in pH from 8 to 6.5) at a higher density (1.5 × 10^5^ cells/cm^2^), which may interfere with its effects; however, we did not observe any apoptosis even under these conditions (data not shown).

A clonogenic assay showed that the cytostatic effect of metformin was transient and that cells treated with metformin for 18 days were still able to form new colonies in complete medium after drug removal. Moreover, “rescued cells” re-treated with metformin recapitulated the growth arrest. HCT116 p53−/− cells had more difficulty in reverting this transient effect, thus suggesting that metformin is more effective in the absence of *TP53* as previously proposed *in vivo* and *in vitro* ^6^. The reversible effect of metformin was highlighted by the reactivation of the mTOR pathway and IGF1R protein after drug removal and three days’ growth in fresh complete medium. Similar results were obtained for p-Rb, cycD1, cycE and c-Myc.

The anti-proliferative action of metformin seems to be mediated by the suppression of the mTOR pathway and IGF1R inactivation^36^, which both play a critical role in cell growth and are often deregulated in patients with various cancers (including CRC), who have high expression levels of these proteins^37^. Metformin reduced the phosphorylation of mTOR, its downstream effectors (ribosome protein S6 and 4EBP1) and IGF1R. Interestingly, the inhibition of mTOR was also observed in the HCT116 and HCT116 p53−/− cells in which AMPK was not activated, thus suggesting that the antiproliferative effect of metformin on CRC cells was not only mediated by AMPK. There is evidence that metformin can inhibit mTOR by means of molecular mechanisms that inhibit the phosphoinositide 3-kinase/Akt/mTOR (PI3K/Akt/mTOR) signalling pathway (such as the inactivation of RAG GTPases), or activate the mTOR inhibitor REDD1 (REgulated in Development and DNA damage responses)^38,39^.

Metformin can also reversibly inhibit complex I of the mitochondrial respiratory chain, an important source of ROS production^3^. ROS are involved in the chemical damage of cell components that also participate in maintaining cell redox homeostasis and signal transduction pathways. ROS play a complex role in cancer, and can promote survival or apoptosis depending on their concentration and the type of cancer cell^40^. It has been suggested that metformin stimulates ROS production, thus providing an alternative mechanism to AMPK activation^41^. We found that ROS production was increased in HCT116 and HCT116 p53−/− cells, but not in HT29 cells, which suggests that metformin may act in these two cell lines by increasing ROS levels and inhibiting mTOR. The finding that an increase in ROS levels induces cell cycle arrest^42^ is in line with our data, and similar findings have been made in ovary^43^ and luminal breast cancer^44^.

The distinctive effects of metformin observed on the cellular models analysed may be due to the different genetic background of the cell lines used in the study. In fact, HCT116 and HCT116 p53−/− cells are KRAS-mutant (G13D), while HT29 cells are BRAF-mutant (V600E). Gain-of-function missense mutations in *KRAS* and *BRAF* genes are mutually exclusive CRC drivers and have similar but subtly different roles during initiation and tumor development. Their mechanistic differences in signal transduction could provide distinct differences in response to therapeutic interference^45^. However, we showed the same effects of metformin, although through different molecular mechanisms, in cells with different genetic driven mutations. These observations support the conclusion of a study investigating the association between metformin use and outcome in patients with stage III CRC in adjuvant chemotherapy who used or not metformin prior to CRC diagnosis, considering also their tumoral KRAS and BRAF status. This study did not find any association between metformin use and disease-free survival, time to recurrence, and overall survival^46^.

Metformin seems to selectively target the CSCs of a number of cancer types^2^, and combined treatment with metformin and chemotherapy can partially affect the viability of CSCs in chemo-resistant CRC cell lines^47^. We found that metformin reduced the RNA expression of two colorectal CSC markers involved in Wnt signalling^32,33^, CD44 and LGR5, thus confirming that it may affect cancer cells with “stem” characteristics. Interestingly, our findings of mitochondrial depolarisation (which was more marked in HCT116 and HCT116 p53−/− cells) and ROS release are in line with the observation that CSCs are more dependent on mitochondrial respiration respect to non CSCs^48^.

Metformin is typically administered at doses of 1 g/day, and its median plasma concentration is 330 μM^49^. The dose used in ours and other *in vitro* studies of CRC^5^ is therefore higher than the therapeutic dose. However, metformin accumulates in tissues at concentrations that are several times those found in blood^49^, and drug concentrations in the gut are higher than in the rest of the body^50^. In addition, CRC cells express high levels of organic cation transporter 1 (OCT1), the main metformin transporter, which may increase the uptake of metformin^51^.

Finally, Dowling *et al*. showed that intracellular accumulation levels of metformin in HCT116 cells were only 10%–15% of the drug present in the medium, and that the lowest dose of metformin required to activate AMPK in cell culture is 1 mM^52^. These observations may justify testing the use of higher metformin concentrations in the treatment of CRC.

## Conclusion

Our findings suggest that metformin can transiently inhibit the growth and proliferation of CRC cells and of their stem fraction by activating AMPK or by promoting oxidative stress, thus suggesting that its use alone is not suitable for cancer treatment. Future studies should consider strategies that synergistically combine metformin with drugs that can benefit from the inhibition of cell growth functions and induce cell death.

## Materials and Methods

### Cell lines and culture conditions

The HT29 and HCT116 cell lines were obtained from the American Type Culture Collection (ATCC), and the HCT116 p53−/− isogenic cells in which the *TP53* gene was inactivated by means of homologous recombination were kindly provided by Professor Bert Vogelstein (Johns Hopkins University, Baltimore, MD, USA). The cells were maintained at 37 °C with 5% CO_2,_ and cultured in McCoy’s 5A (modified) medium, GlutaMAX^TM^ supplement (ThermoFisher Scientific, Inc., Waltham, MA, USA) plus 10% fetal bovine serum (FBS) and 1% penicillin-streptamycin (ThermoFisher Scientific, Inc.).

### Chemicals

Metformin (Sigma-Aldrich, St. Louis, MO, USA) was diluted in sterile water to make the 1 M stock solution that was used at a concentration of 5 mM in all of the experiments, with the exception of migration and invasion assays, in which scalar concentrations, ranging from 5 to 0.3 mM were tested.

### Immunoblotting

The cells were lysed in Ripa buffer (50 mM Tris, pH 8.0, 50 mM NaCl, 0.5% Triton X-100, 0.1% sodium deoxycholate, 0.25% sodium dodecyl sulphate [SDS]) supplemented with protease inhibitors (Merck Millipore, Billerica, MA, USA) and a phosphatase inhibitor cocktail (Sigma-Aldrich, St. Louis, MO, USA), and their protein content was quantified using the BCA protein assay (ThermoFisher Scientific, Inc.). Cells treated with metformin for 72 hours, left untreated, or grown for 72 hours with metformin and for a further 72 hours in fresh complete medium without the drug (rescued cells) were used. For each sample, 40 μg of protein extract were separated on 4–12% polyacrylamide gels, transferred onto nitrocellulose membranes (Sigma-Aldrich, St. Louis, MO, USA), and incubated with primary antibodies (see Supplementary Table S1). The signals were detected using enhanced chemiluminescence, and protein levels were quantified using Imagelab^TM^ software (Bio-Rad, Hercules, CA, USA). Each experiment was repeated at least three times.

A densitometry scan of the bands was performed to quantify the results. Finally data analysis was done by Image J software (NIH, Bethesda, MD), measuring integrated densities of obtained bands after background subtraction.

### Cell proliferation assay by means of 5-bromo-2′-deoxyuridine (BrdU) incorporation

One thousand cells were seeded on 13 mm diameter coverslips (ThermoFisher Scientific, Inc.) and, after 24 hours, metformin was added for 72 hours. The cells were incubated with 33 μM BrdU (Sigma-Aldrich), anti-BrdU primary antibody (BD Biosciences, Franklin Lakes, NJ, USA) and secondary FITC-conjugated antibody (Jackson ImmunoResearch, West Grove, PA, USA). Their nuclei were visualised using 4′, 6-diamidino-2-phenylindole (DAPI; Sigma-Aldrich), and the images were acquired using an upright, automated Olympus BX-61 microscope (Shinjuku, Tokyo, Japan).

### Annexin V assay

Cell apoptosis was evaluated by Annexin V assay. The cells were seeded in a 35 mm multi-well plates (2 × 10^5^ cells per well) and, after 24 hours, were treated with metformin for 72 hours. They were then harvested, washed once with Annexin buffer (1 M HEPES, 5 M NaCl, 1 M MgCl_2_, 1 M CaCl_2_, 50 mM KCl) and stained with anti-Annexin V-APC conjugated primary antibody (BD Biosciences, Franklin Lakes, NJ, USA). Then the cells were washed once with Annexin buffer, stained with propidium iodide and analysed using a FACSCanto II system (BD Biosciences) and ModFit LT 3.0 software. Three independent experiments were carried out. Cells treated with 20 mM Metformin were used as control of apoptosis induction (Supplementary Fig. S5).

### Cell motility assay

Cell migration was evaluated using a modified wound scratch assay. The cells were plated (5 × 10^4^ for HT29; 3 × 10^4^ for HCT116; 4 × 10^4^ for HCT116 p53−/− cells) in a cell culture dish (Ibidi, Martinsried, Germany) and, when cell confluence was approximately 90%, the chambers were removed and the cells were treated with metformin. The images (magnification 10x) were acquired immediately after removing the chambers, and at the complete closure of the gap in the untreated cells using an inverted EVOSmicroscope (Thermofisher Scientific, Inc., Waltham, MA, USA).

Cell invasion was evaluated by plating 3 × 10^4^ cells per well in a 24-well BDBioCoat growth factor-reduced MATRIGEL invasion chamber (BD Biosciences, Franklin Lakes, NJ, USA), and treating them with metformin. Inserts were placed in a well containing 750 µl of normal growth medium with 10% FBS. After 72 hours (HCT116 and HCT116 p53−/− cells) or 96 hours (HT29 cells), the cells in the upper compartment were removed, and the invading cells attached to the bottom side were fixed with cold methanol and stained with Crystal Violet (Sigma-Aldrich). Three independent experiments were carried out.

### Cell cycle analysis

Cells were plated in 10 mm Petri dishes (5 × 10^5^ cells/well) and, after 24 hours, were treated with metformin for 72 hours. They were then scraped, fixed in ice-cold 70% ethanol, and stained in accordance with the RNAse/propidium iodide protocol. Cell cycle distribution was analysed using a FACSCanto II system (BD Biosciences) and ModFit LT 3.0 software.

### Immunohistochemistry (IHC)

HT29 and HCT116 cells were detached from plates using 1% trypsin, washed twice with PBS, fixed using 10% PBS-buffered formalin, and then inserted in 500 µl of pre-heated (60 °C) 2% agar solution (Cat. No. 50005, Lonza, Basel, Switzerland). After agar solidification, the cells were embedded in paraffin using standard procedures. The IHC experiments were carried out using 3 µm cell block sections. C-Myc (Cat. No. 32072, Abcam, Cambridge, UK) and phospho-histone H3 (Ser10, Cat. No. #9701, Cell Signaling Technology, Danvers, MA, USA) were respectively diluted 1:50 and 1:200, and antigen retrieval and development were carried out using a fully automated BenchMark ULTRA (Ventana Medical Systems, Inc., Tucson, AZ, USA) instrument in accordance with the manufacturer’s instructions.

### *In situ* hybridisation (ISH)

ISH for leucine-rich repeat-containing G-protein coupled receptor 5 (LGR5) RNA expression was carried manually using the RNAScope 2.0 High Definition (Brown) detection kit (Advanced Cell Diagnostics, Inc., Hayward, CA, USA) in accordance with the manufacturer’s instructions, as previously described^53^.

### Senescence-associated (SA) β-galactosidase activity

The cells were seeded in 35 mm multi-well plates (3.75–30 × 10^4^ cells/well) and then treated with metformin for 72 hours. SA-β-galactosidase activity was detected using the Senescence β-galactosidase staining kit (Cell Signaling Technology, Inc.) in accordance with the manufacturer’s instructions. The cell images were acquired under reflected light using a motorised inverted Olympus IX81 microscope. The cells used as positive controls were obtained as previously described^54^.

### Colony-formation assay

The cells were seeded in 35 mm multi-well plates (10^2^ cells/well) and treated with metformin for 6, 12 and 18 days. The medium was refreshed every three days. In order to obtain the rescued cells, the drug was removed and the cells were allowed to grow for 6, 12 and 18 days in fresh medium. The rescued cells were seeded in 35 mm multi-well plates (10^2^ cells/well) and treated with metformin, or left untreated, for 12 days. At the end of the treatments, the plates were stained with Crystal Violet. Only the clearly visible colonies were counted. The experiment was repeated at least three times with three replicates every time.

### RNA extraction and quantitative real-time PCR (qRT-PCR)

Total RNA was extracted from the cells using the Trizol reagent (Invitrogen, Carlsbad, CA, USA) in accordance with the manufacturer’s protocol. TaqMan gene expression assays were used for qRT-PCR as previously described^55^, and the expression revealed by each assay (MMP9 Hs00234579_m1, LGR5 Hs00173664_m1 and CD44 Hs00153304_m1) was normalised to that of GAPDH (Hs99999905_m1). The data were analysed using Sequence Detector, version SDS 2.1.

### Reactive oxygen species (ROS) detection assay

The cells were seeded in 96-well microplates (1.5 × 10^3^ cells/well) and, after 24 hours, treated with metformin. Hydrogen peroxide (H_2_O_2_) levels were measured using the ROS-Glo^TM^ H_2_O_2_ assay (Promega, Fitchburg, WI, USA) in accordance with the manufacturer’s instructions. The bioluminescent readings were obtained from three independent experiments using a microplate reader (TECAN Group Ltd., Männedorf, Switzerland).

### Mitochondrial membrane potential (Δψ_m_) analysis

The mitochondrial membrane potential of the cells was detected using a MitoProbe™ JC-1 (5,5′,6,6′-tetrachloro-1,1′,3,3′-tetraethylbenzimidazolylcarbocyanine iodide) Assay Kit for flow cytometry (ThermoFisher Scientific, Inc.) in accordance with the manufacturer’s instructions. The cells were treated with metformin for 72 hours, and analysed using a FACSCanto II system. Before harvesting the cells, 1 μl of carbonyl cyanide 3-chlorophenylhydrazone (CCCP) was added as a positive control.

### Statistical analysis

The data are presented as mean values ± standard deviation (SD) and statistically compared between groups using one-way analysis of variance followed by Student’s t-test. A P-value of <0.05 was considered statistically significant.

## Electronic supplementary material

Supplementary Information
