# Supplementary material for: Metformin transiently inhibits colorectal cancer cell proliferation as a result of either AMPK activation or increased ROS production
Source: Sci Rep. 2017 Nov 22;7:15992. doi: 10.1038/s41598-017-16149-z (PMC5700100; doi:10.1038/s41598-017-16149-z)
Supplement: Supplementary file 1 — Supplementary Information [file 41598_2017_16149_MOESM1_ESM.pdf]

SUPPLEMENTARY INFORMATION

Metformin transiently inhibits colorectal cancer cell proliferation as a result of either AMPK activation or increased ROS production

Angela Mogavero[1,2], Maria Valeria Maiorana[1,2], Susanna Zanutto[1,2], Luca Varinelli[1,2], Fabio Bozzi[3], Antonino Belfiore[3], Chiara C. Volpi[3], Annunziata Gloghini[3], Marco A. Pierotti[2], Manuela Gariboldi[1,2,*]

SUPPLEMENTARY MATERIALS AND METHODS

Cell viability assay (MTT)

The cells were seeded at a density of 1.5×10^3 into 96-well microplates. A standard MTT assay was conducted after 72 hours of treatment with metformin (concentration range 1.25–80 mmol/L). The absorbance was measured with a microplate reader (Infinite 200, TECAN Group, Ltd., Männedorf, Switzerland) at 595 nm.

Wound healing assay

A wound healing scratch assay was performed to evaluate the effects of metformin on cell migration. The cells were seeded in a 35 mm multi well plates (2×10^5 cells per well for HCT116 and HCT116 p53-/- cell lines, and 2.5×10^5 cells per well for HT29 cells). When cells confluence was approximately 90%, a manual scratch was performed using a sterile p200 tip. The cells were then treated with scalar concentrations of metformin, ranging from 5 to 0.3 mM. The images (magnification 4X) were acquired immediately after the scratch (0h) and at the complete closure of the gap in the untreated cells (96 hours for HT29, 72 hours for HCT116 and HCT116 p53-/-), using an inverted EVOSmicroscope (ThermoFisher Scientific, Inc., Waltham, MA, USA).

Caspase-3 assay

The cells were seeded at a density of 5×10^5 cells and after 24 hours treated with metformin for 72 hours. The cells were harvested, fixed in 1% formaldehyde and left in ice-cold 70% ethanol. The cells were incubated with anti-Caspase-3 antibody (Cell Signaling Technology, Danvers, MA, USA), then with a secondary FITC-conjugated antibody (Cell Signaling Technology) and analysed on a FACSCanto II.

Immunofluorescent (IF) staining and image analysis for autophagy

The cells were seeded at a density of $2.5–10\times10^4$ directly on a 13 mm diameter coverslip and treated with metformin for 72 hours. The cells were fixed in 4% paraformaldehyde and incubated with anti-LC3B antibody. To visualize the nuclei, the cells were treated with DAPI. Images were acquired with Olympus BX-61 automated upright microscope.

Human cervical adenocarcinomas cells (Hela), obtained from ATCC, were used as controls. The cells were maintained at 37°C with 5% CO_2 and cultured in Minimum Essential Medium (MEM; ThermoFisher Scientific, Inc., Waltham, MA, USA) supplemented with 10% FBS and 1% Penicillin-Streptamycin. For IF, the cells were seeded at a density of 12.5×10^4 on a 13 mm

diameter coverslip and treated with 50 µM N^4-(7-Chloro-4-quinolinyl)-N^1,N^1-dimethyl-1,4-pentanediamine (chloroquine diphosphate salt; Sigma-Aldrich, St. Louis, MO, USA) for 24 hours.

Erythrosine B exclusion assay

A standard erythrosine B exclusion assay was used to evaluate cell viability after metformin treatments. The cells were seeded in 100 mm Petri dish (8^4 cells/Petri) and treated with metformin for 3, 6 and 12 days. The medium was refreshed every three days. At the end of the treatments the cells were stained with erythrosine B and counted with a Burker chamber. To obtain the rescued cells, the drug was removed and cells were allowed to grow for 6 days in fresh medium. Rescued cells were plated in a 100 mm Petri dish (8^4 cells/Petri), re-treated with metformin for 3, 6 and 12 days and counted after erythrosine B staining. The experiment was repeated at least three times with three replicates every time. Data were analysed using the Student's *t*-test.

Cell proliferation curves

Cell proliferation curves were obtained by seeding the cells in a 100 mm Petri dish (8^4 cells/Petri). After 24 hours the cells were treated with metformin for 3, 6 and 12 days. The medium was refreshed every three days and at the end of the treatments the total number of cells was determined counting with the Burker chamber. The rescued cells were obtained allowing them to grow in a fresh medium for 6 days after metformin treatment. After that, rescued cells were re-treated with metformin and counted as above. The experiment was repeated at least three times with three replicates every time. Data were analysed using the Student's *t*-test.

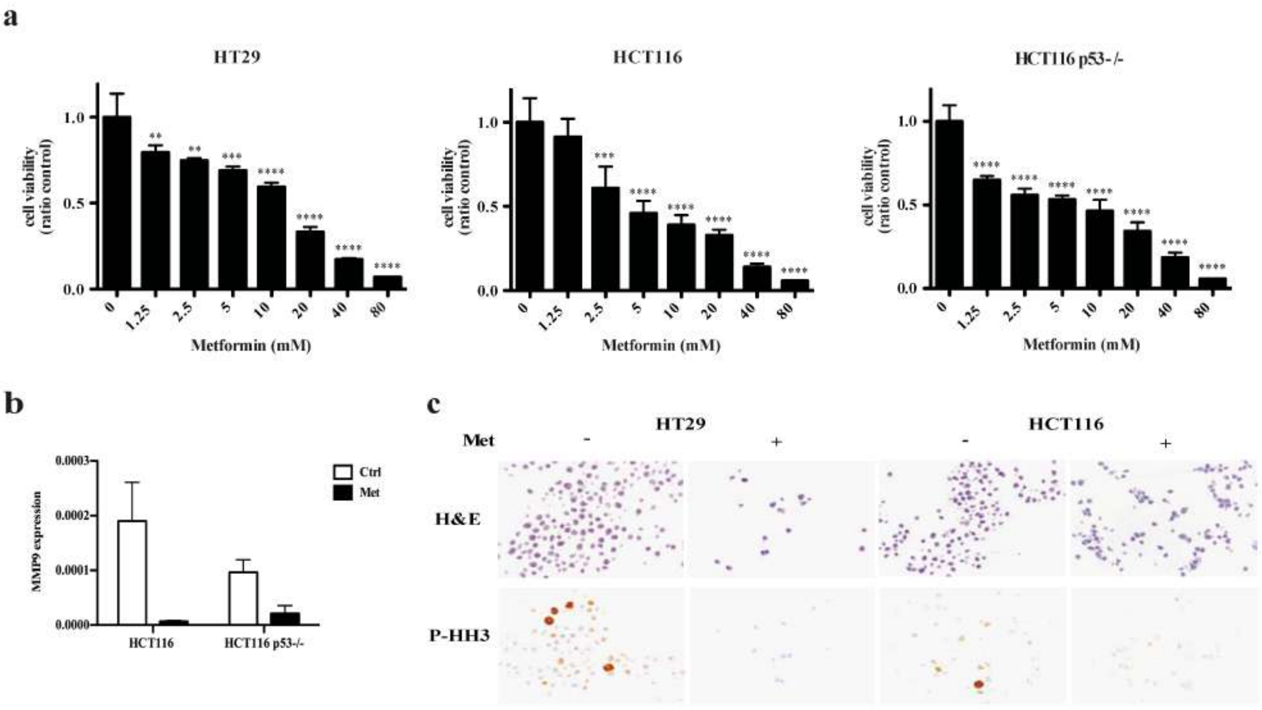

Supplementary Figure S1. Metformin (Met) inhibits the proliferation and invasion of CRC cells. a) Cell viability was expressed as the percentage of untreated cells. The bars represent the mean values ± SD of three independent experiments (**P<0.01, ***P<0.001, ****P<0.0001). b) qRT-PCR evaluation of MMP9 expression in HCT116 and HCT116 p53-/- cells before (white) and after Met treatment (black). The qRT-PCR data are shown as delta cycle threshold value ($2^{-\Delta Ct}$), which directly correlated with gene expression levels in each sample. c) Immunohistochemistry of histone H3 phosphorylation (P-HH3) in HT29 and HCT116 cells.

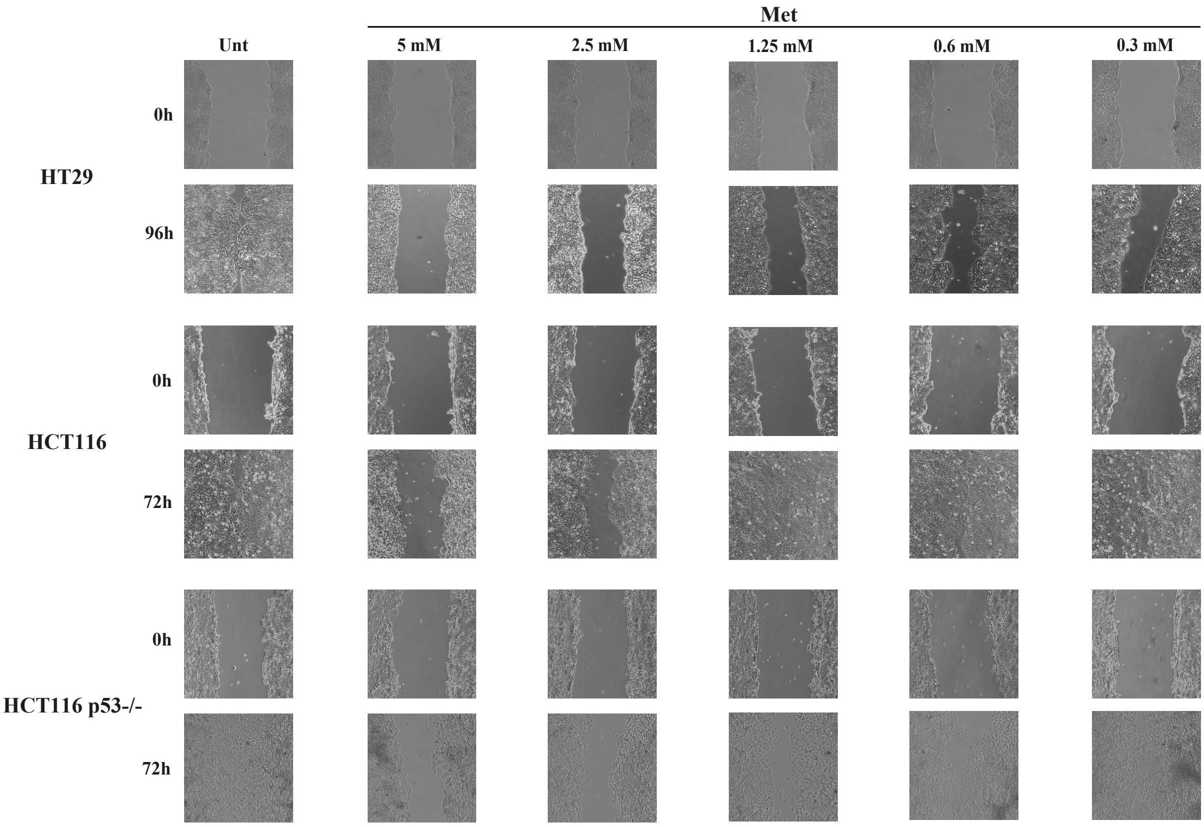

Supplementary Figure S2. Metformin (Met) decreased the migration of HT29, HCT116 and HCT116 p53-/- cells. A manual wound-healing scratch assay was performed after treatment with scalar concentrations of Met (ranging form 5 to 0.3 mM) for 96h (HT29) or 72h (HCT116 and HCT116 p53-/-).

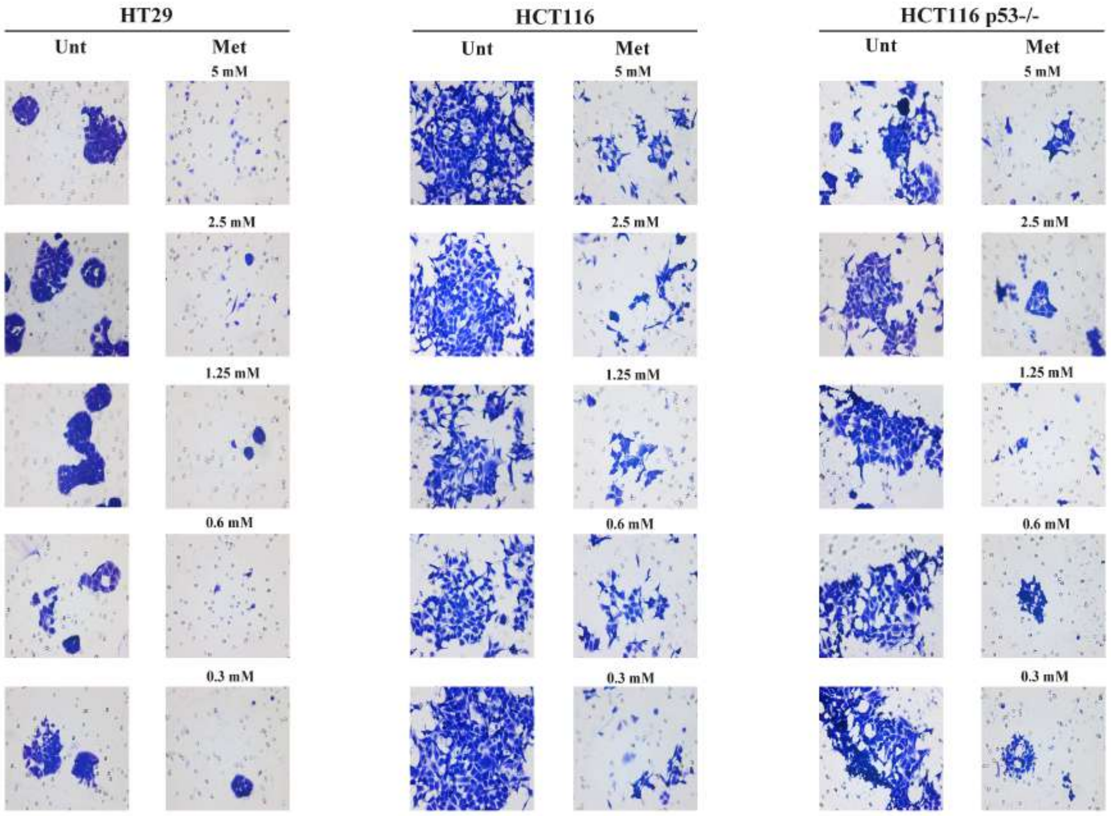

Supplementary Figure S3. Metformin (Met) decreased the invasion of HT29, HCT116 and HCT116 p53-/- cells. The chamber invasion assay was performed after treatment with scalar concentrations of Met (ranging from 5 to 0.3 mM) for 96 hours (HT29 cells) or 72 hours (HCT116 and HCT116 p53-/- cells).

a

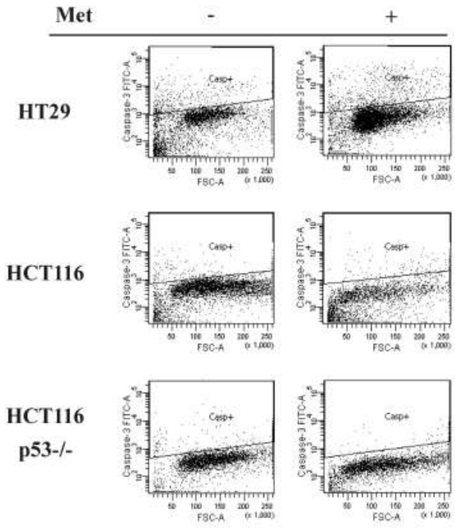

b

Cell line	# of cells	# of Casp+ cells	% of Casp+ cells
HT29 Unt	9,100	403	4.42
HT29 Met	46,217	1,980	4.28
HCT116 Unt	21,112	439	2.08
HCT116 Met	9,256	146	1.58
HCT116 p53-/- Unt	40,260	643	1.60
HCT116 p53-/- Met	42,791	152	0.36

c

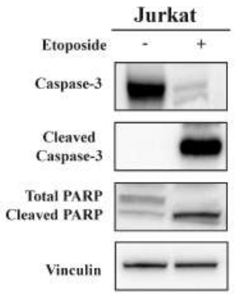

Supplementary Figure S4. Metformin (Met) does not induce apoptosis or autophagy in HT29, HCT116 or HCT116 p53-/- cells. a) Flow cytometric detection of cells containing active Caspase-3 after 72 hours' treatment with Met or not. b) The table shows the total and the active Caspase-3 positive cells (Casp+) detected by flow cytometry. c) Jurkat's lysate treated with etoposide was used as positive control for Caspase-3 and PARP activation. Vinculin was used as the loading control. The data are representative of three independent experiments.

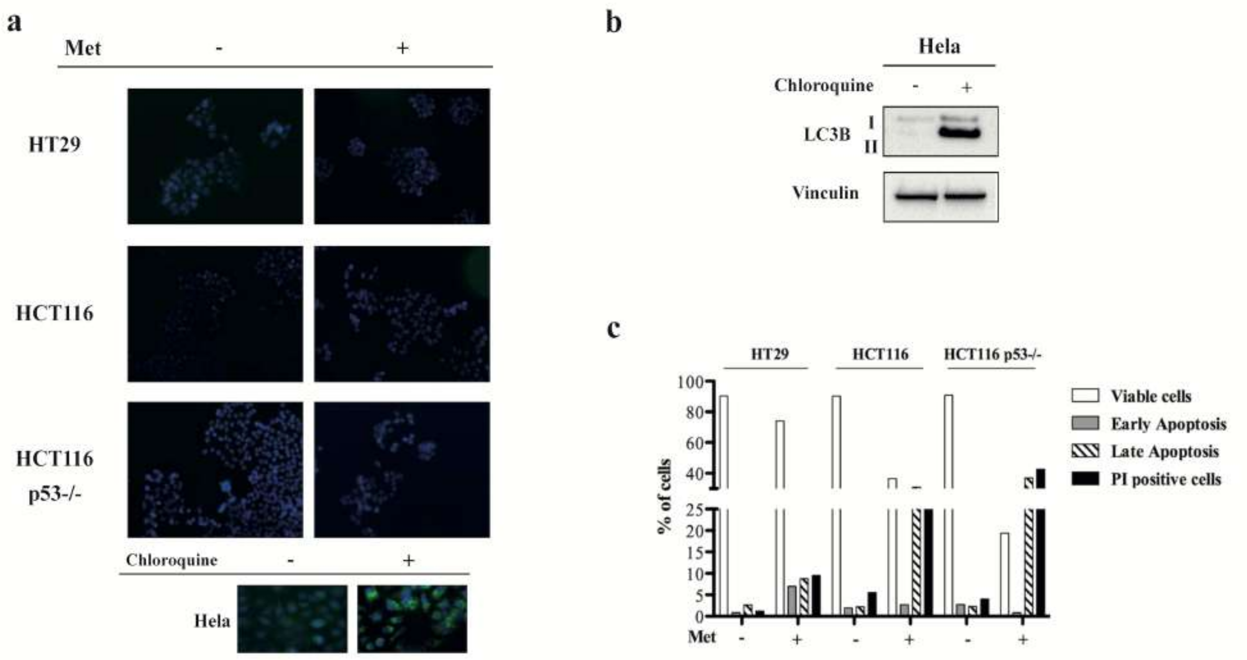

Supplementary Figure S5. Metformin (Met) does not induce apoptosis or autophagy in HT29, HCT116 or HCT116 p53-/- cells. a) Immunofluorescence analysis of LC3B expression (magnification 20x). At the bottom, Hela cells left untreated or treated with chloroquine for 24 hours and used as positive controls (magnification 40x). b) Hela's lysate treated with chloroquine was used as positive control for autophagy activation. Vinculin was used as the loading control. All data are representative of three independent experiments. c) Annexin V assay in cells treated for 72 hours with 20 mM Met or left untreated, used as positive control of apoptosis induction.

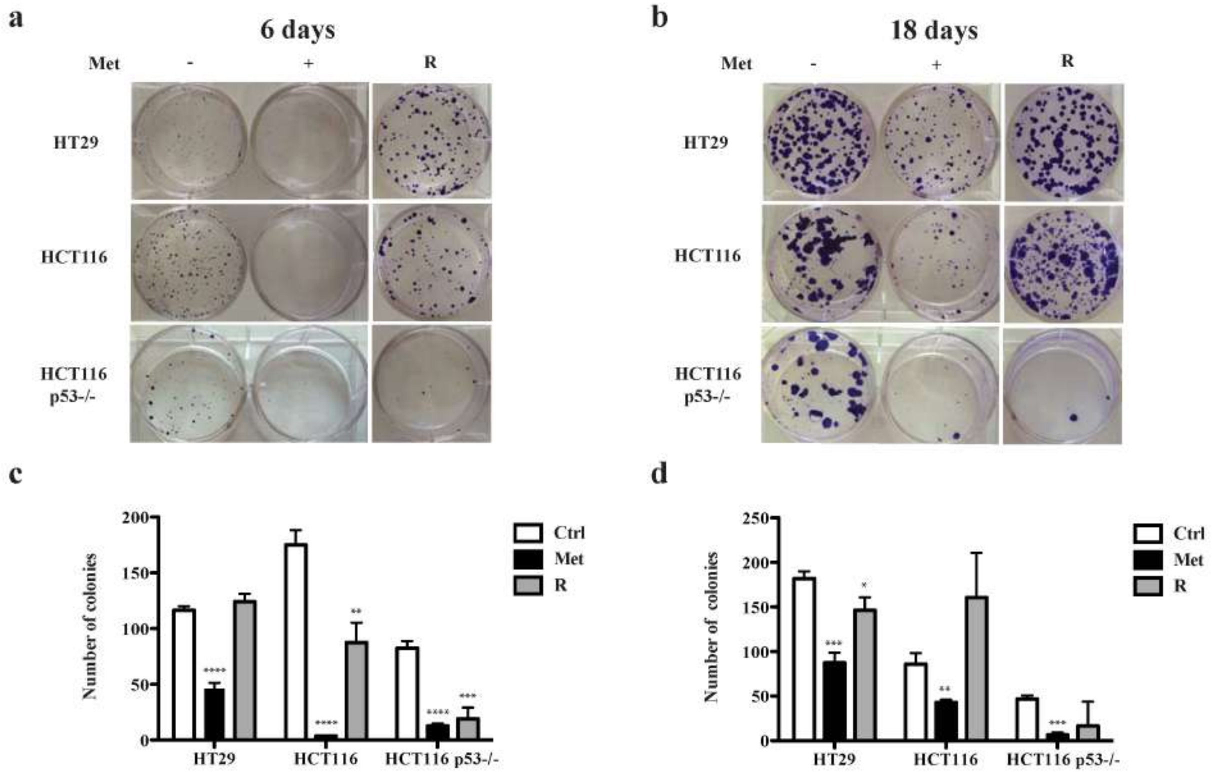

Supplementary Figure S6. Metformin (Met) inhibits colony formation and this effect is reversible. The cells were grown for six (a) or 18 days (b) in the presence of Met. The rescued colonies (R) were treated with Met for six or 18 days, and grown for a further six or 18 days in fresh complete medium without Met. c, d) Graphic representations of the results. The bars represent the mean value ± SD of three independent experiments (*P<0.05, **P<0.01, ***P<0.001, ****P<0.0001).

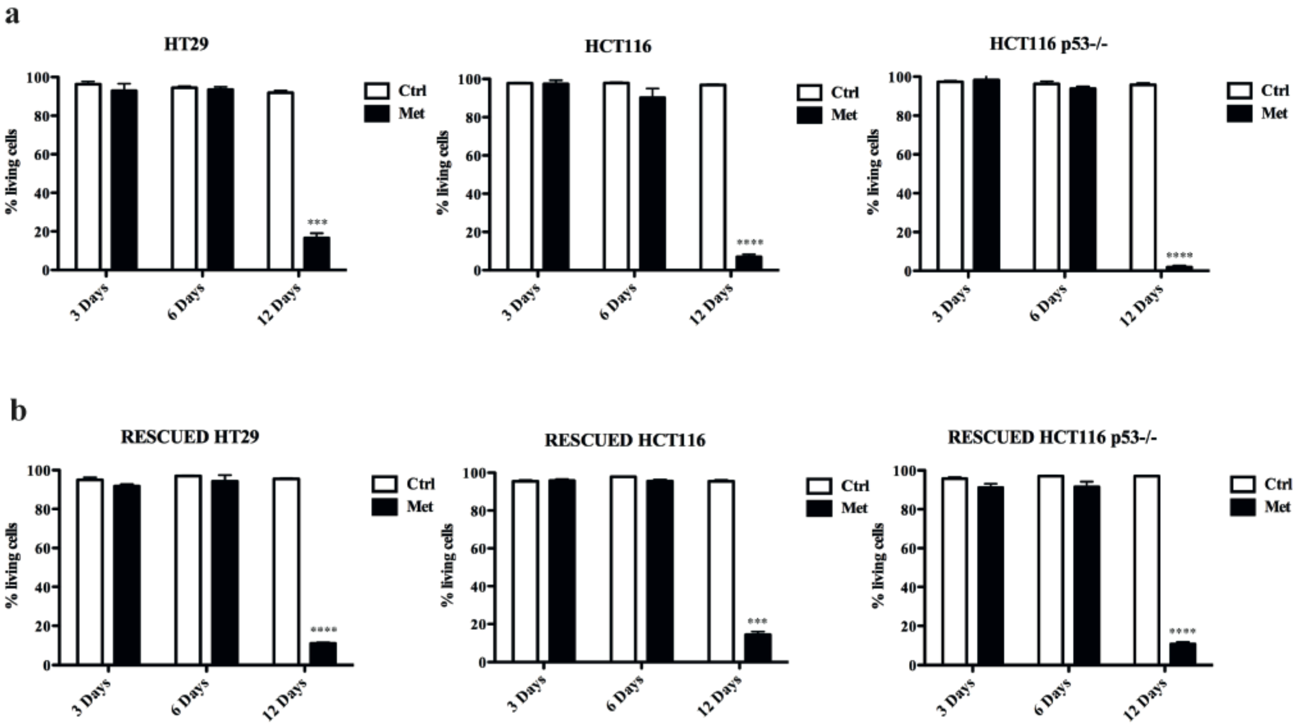

Supplementary Figure S7. Viability of metformin (Met) treated cells is comparable to that of rescued cells re-treated with the drug. a) The cells were treated with Met, or left untreated, for 3, 6 and 12 days. At the end of each treatment the cells were stained with erythrosine B and counted with a Burker chamber. b) The Rescued cells were grown in a fresh medium for 6 days after Met treatment, seeded in a 100 mm Petri dish (8^4 cells/Petri), re-treated with Met for 3, 6 and 12 days, or left untreated, and counted after erythrosine B staining. The bars represent the mean value ± SD of three independent experiments (***P<0.001, ****P<0.0001).

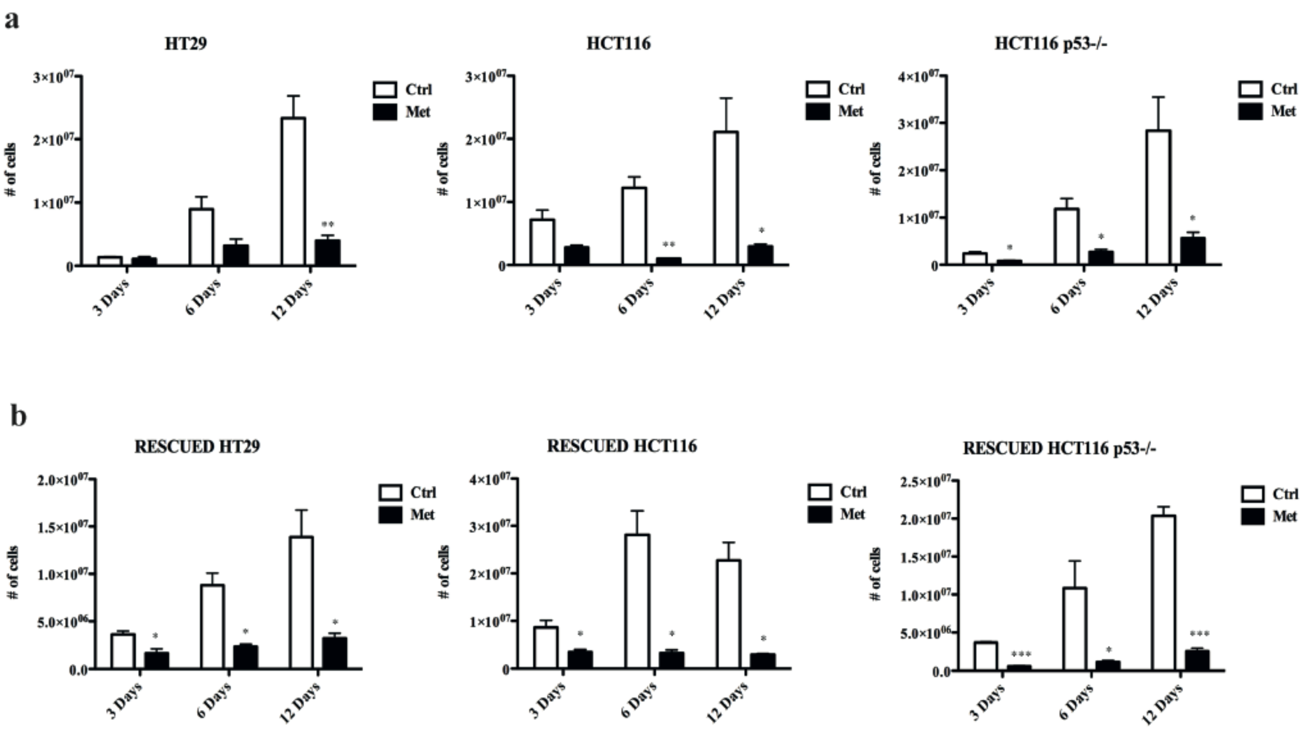

Supplementary Figure S8. Proliferation of metformin (Met) treated cells is comparable to that of rescued cells re-treated with the drug. a) The cells were treated with Met, or left untreated, for 3, 6 and 12 days and counted with a Burker chamber at the end of each treatment. b) The Rescued cells were grown in a fresh medium for 6 days after Met treatment, seeded in a 100 mm Petri dish (8^4 cells/Petri), re-treated with Met for 3, 6 and 12 days, or left untreated, and counted. The bars represent the mean value ± SD of three independent experiments (*P<0.05, **P<0.01, ***P<0.001).

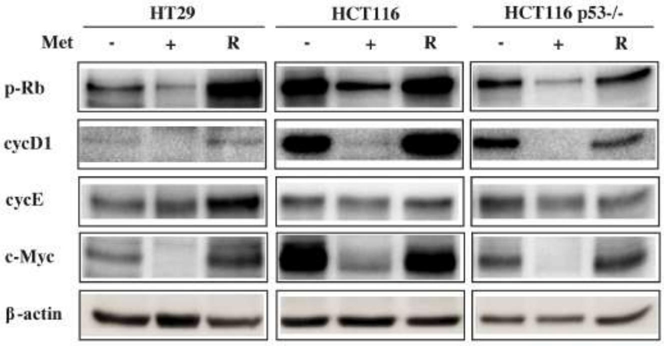

Supplementary Figure S9. Metformin (Met)-induced inhibition of proliferation is reversible. Immunoblots of p-Rb, cycD1, cycE and c-Myc in cells treated for 72 hours with 5 mM Met or left untreated. The "Rescued" cells (R) were grown for 72 hours with Met and for a further 72 hours in fresh complete medium without Met. β-actin was used as the loading control. The results are representative of three independent experiments.

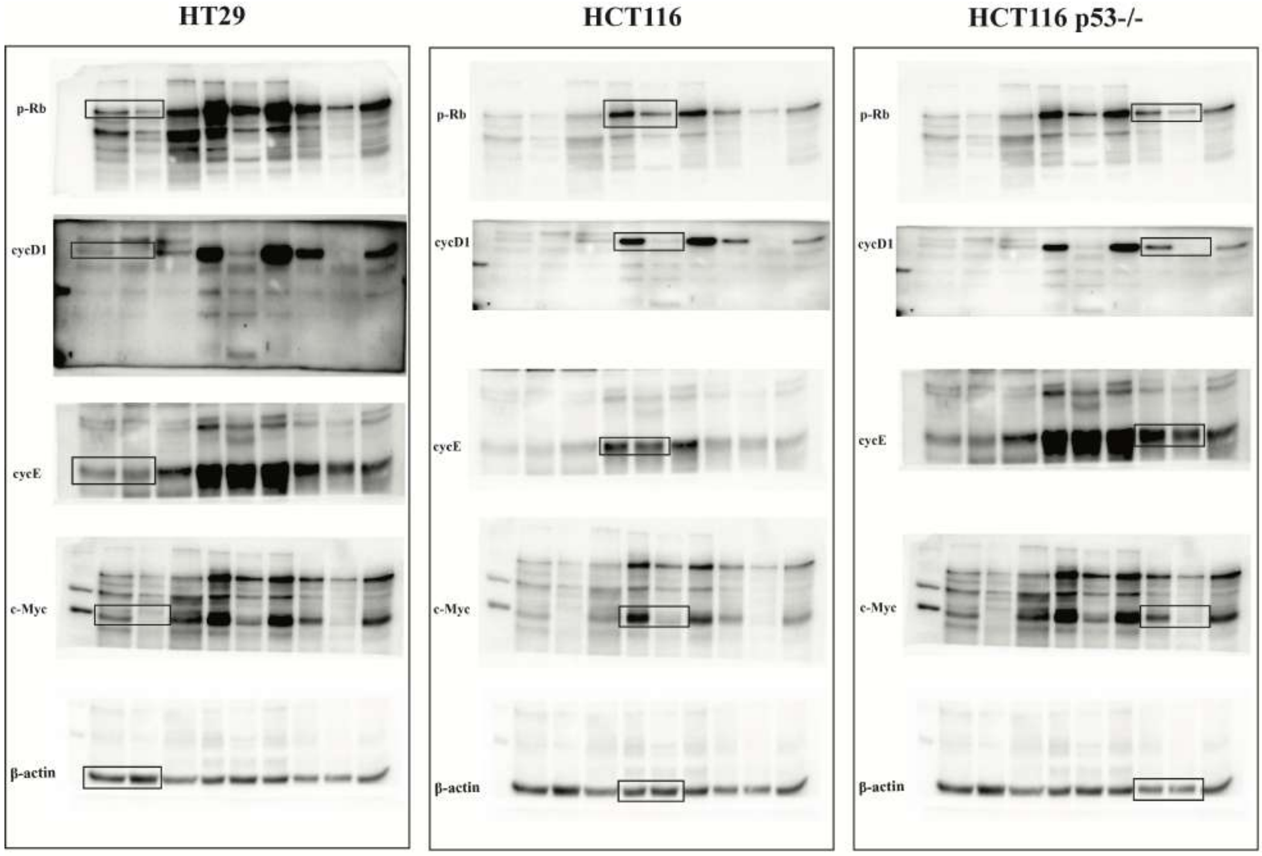

Supplementary Figure S10. Full-size blots of Figure 2b.

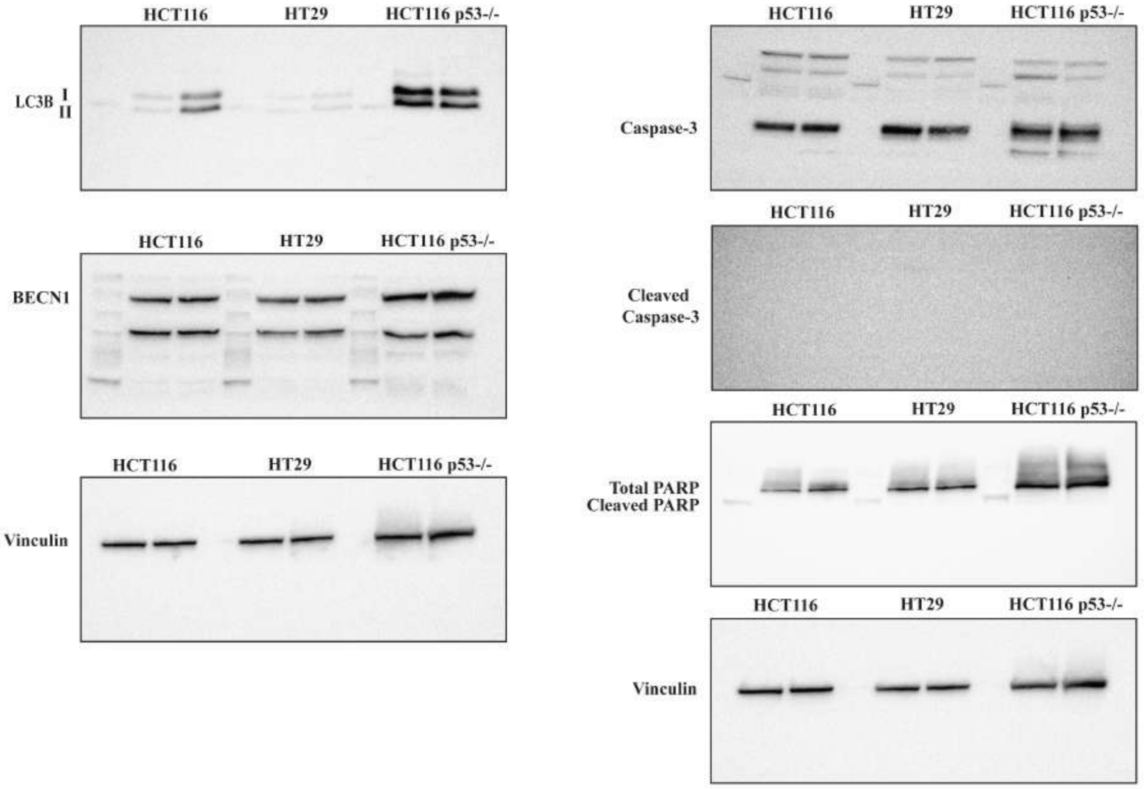

Supplementary Figure S11. Full-size blots of Figures 3a and 3b.

HT29

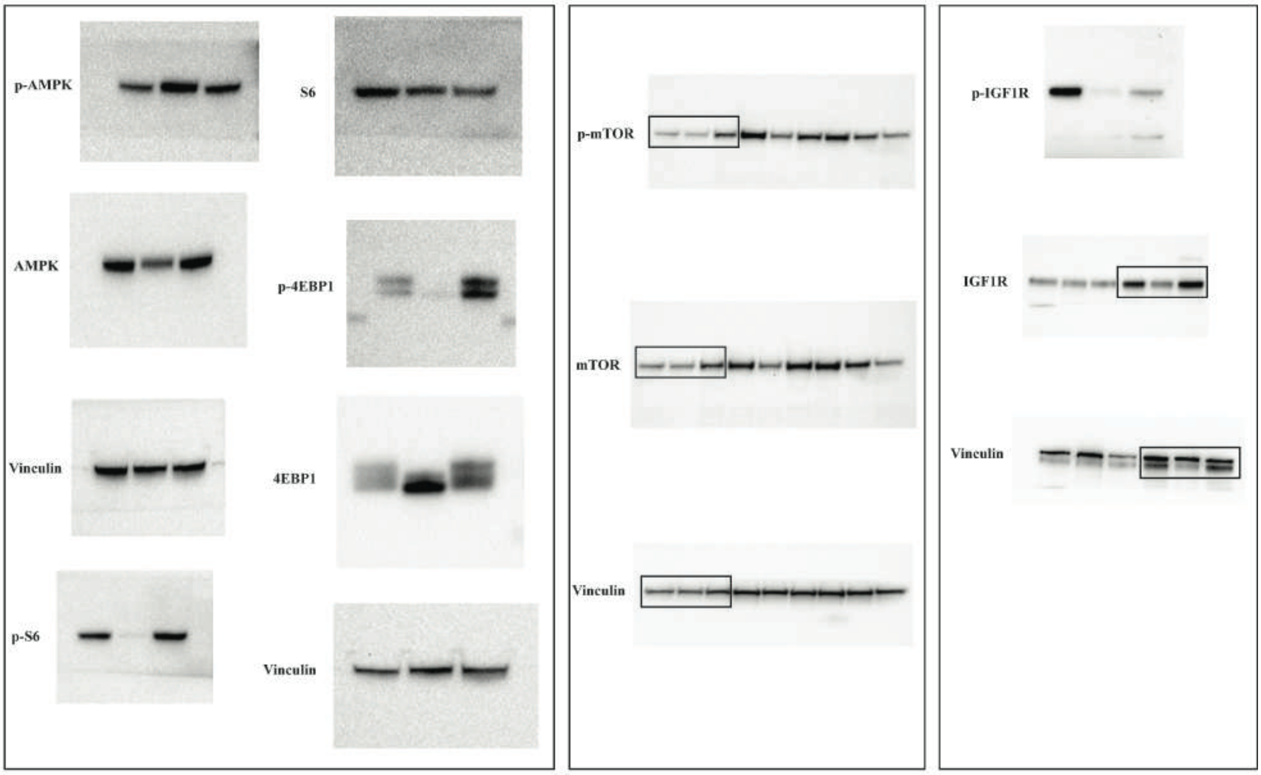

Supplementary Figure S12. Full-size blots of HT29 reported in Figure 6.

HCT116

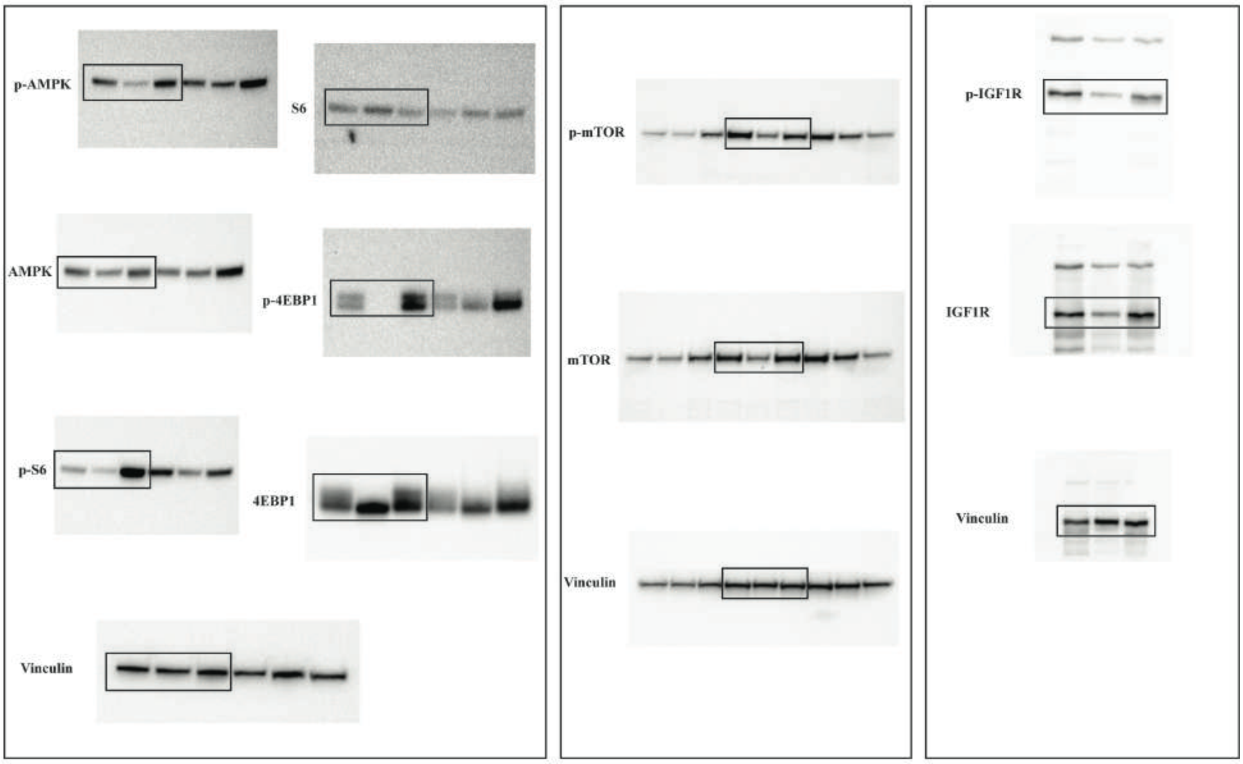

Supplementary Figure S13. Full-size blots of HCT116 reported in Figure 6.

HCT116 p53-/-

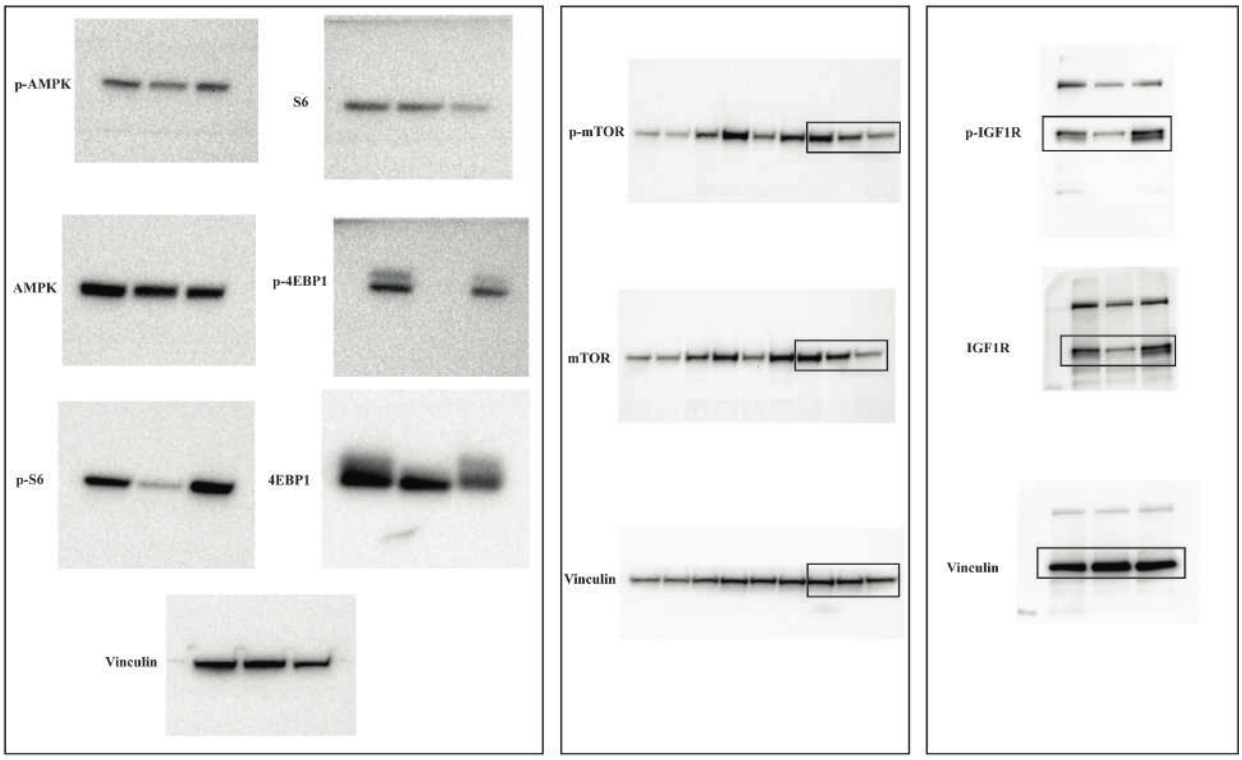

Supplementary Figure S14. Full-size blots of HCT116 p53-/- reported in Figure 6.

Supplementary Table S1. The antibodies used for the immunoblotting analyses.

Antibody	Source	Dilution	Company	Catalog number
β–Actin	Rabbit	1:5000	Sigma-Aldrich (St Louis, MO, USA)	A2066
AMPKα IgG2b	Mouse	1:1000	Cell Signaling Technology (Danvers, MA, USA)	2793
phospho-AMPKα (Thr172) IgG	Rabbit	1:1000	Cell Signaling Technology	2535
cyclin D1	Rabbit	1:500	Santa Cruz Biotechnology (Dallas, TX, USA)	sc-753
cyclin E1 IgG1	Mouse	1:1000	Cell Signaling Technology	4129
4E-BP1 IgG	Rabbit	1:1000	Cell Signaling Technology	9644
phospho-4E-BP1 (Thr70) IgG	Rabbit	1:1000	Cell Signaling Technology	9455
IGF-1 Receptor β	Rabbit	1:1000	Cell Signaling Technology	3027
phospho-IGF-1 Receptor β	Rabbit	1:1000	Cell Signaling Technology	3024
LC3B	Rabbit	1:1000	Cell Signaling Technology	2775
mTOR	Rabbit	1:1000	Cell Signaling Technology	2972
phospho-mTOR (Ser2448)	Rabbit	1:1000	Cell Signaling Technology	5536
c-Myc	Rabbit	1:1000	Cell Signaling Technology	5605
PARP	Rabbit	1:1000	Cell Signaling Technology	9542
phospho-Rb (Ser780) IgG	Rabbit	1:1000	Cell Signaling Technology	3590
S6 IgG1	Mouse	1:1000	Cell Signaling Technology	2317
phospho-S6 (Ser240/244) IgG	Rabbit	1:1000	Cell Signaling Technology	2215
BECN1 (H-300)	Rabbit	1:700	Santa Cruz Biotechnology	11427
Cleaved Caspase-3 (Asp 175)	Rabbit	1:1000	Cell Signaling Technology	9661
Caspase-3	Rabbit	1:1000	Cell Signaling Technology	9662
Vinculin	Mouse	1:5000	Sigma-Aldrich	V9131
Horseradish peroxidase (HRP)-conjugated anti-mouse IgG	Goat	1:8000-1:15000	Bio-Rad (Hercules, CA, USA)	1706516
Horseradish peroxidase (HRP)-conjugated anti-rabbit IgG	Goat	1:8000-1:15000	Bio-Rad	1706515
